# Supplementary material for: Mass extinctions drove increased global faunal cosmopolitanism on the supercontinent Pangaea
Source: Nat Commun. 2017 Oct 10;8:733. doi: 10.1038/s41467-017-00827-7 (PMC5635108; doi:10.1038/s41467-017-00827-7)
Supplement: Supplementary file 1 — Supplementary Information [file 41467_2017_827_MOESM1_ESM.pdf]

Supplementary Note 1: Phylogeny and taxonomy

A time-calibrated informal species-level amniote supertree, including 1046 taxa, was constructed by hand based on the most up-to-date phylogenetic analyses available for Permian-Triassic amniotes. The interrelationships shown in the supertree reflect congruence between overlapping topologies of the source phylogenetic analyses; polytomies were generated when source phylogenies showed incongruent relationships (cf. the semi-strict supertree method automatized by the program TNT; [1]). This tree is given as Supplementary Data 1. This supertree includes 891 species within the interval from the Lopingian to the end of the Early Jurassic. As terrestrial biogeographic patterns were of interest, marine taxa were omitted; however, amphibious non-crocopod archosauromorphs and volant pterosaurs known from marine strata were included in the analysis.

To avoid artificially truncating branch lengths, and so the phylogenetic distances between taxa, 155 stratigraphically older taxa were also included in order to date deeper nodes. This topology was used to produce 100 time-calibrated trees, in which polytomies were randomly resolved, utilizing the 'timePaleoPhy' function of the paleotree package [2] in R [3]. Trees were dated according to first occurrence dates with a minimum branch-length of 1 Myr. The phylogenetic biogeographic analyses were performed across all of these trees, in order to account for phylogenetic uncertainty.

The informal supertree was constructed from pre-existing phylogenies, as described below.

Parareptilia

Overall parareptile phylogeny primarily follows [4–11] with "nycteroleter" relationships following [12–14]. Pareiasaur taxonomy and relationships primarily follow [15], with additional input from [8,13,14]. The two unnamed pareiasaur species known from the d'Ikakern Formation, Argana Basin, of Morocco are placed following [16]. *Sauropareia* and *Colletta* are placed as

successive outgroups of the Procolophonidae following [17]. Procolophonid relationships then follow [4,7,8,18–20], although conflicts between analyses and incomplete taxon sampling required the collapse of many nodes. Additional input on procolophonid taxonomy was taken from [21,22]. *Libognathus* was considered to lie proximate to *Leptopleuron* and *Hypsognathus* following the comparisons of [23,24]. *Haligonia* exhibits a greatly expanded maxillary tooth otherwise only known from *Phonodus* [25]; consequently it was treated as the sister-taxon to *Phonodus* here based on the comparisons of [25]. *Orenburgia* and *Procolina* were placed in a polytomy with the other "kapoids" *Anomoiodon* and *Kapes* following the comparisons of [26].

Eureptilia

Overall eureptile topology follows [27,28]. Captorhinid relationships follow [29]. *Gecatogomphius* was considered a moradosaurine after [30]. Early diapsid phylogeny then follows [27,28]. Drepanosaurids were positioned as the sister group to coelurosauravids after [31]; internal relationships of the clade follow [32,33]. *Palaeagama* was placed following [34] and *Saurosteon* after [35].

Lepidosauromorpha – *Paliguana* is positioned basal to [Kuehneosauridae + Lepidosauria] after [36]. Kuehneosaurid relationships follow [26,37]. Stem-lepidosaur relationships follow [26,35,36,38,39]. *Tikiguana* is probably Cenozoic in age [40] and most purported stem-squamates from this interval are either very poorly phylogenetically constrained, or probable procolophonians [21,36,41]. This leaves *Paikasaurus* and *Bharatagama* as the only named squamates recognized from the interval of interest [36,38,42].

Rhynchocephalian topology follows [38,43–47], although topological conflict and incomplete taxonomic sampling limited phylogenetic resolution. *Sigmala* was considered a sphenodontid due to its similarity to taxa such as *Pelecymala* [48]. "Clevosaur" taxonomy and relationships follow [49], although taxonomic uncertainty, the inclusion of poorly-known but

biogeographically important specimens (e.g. the Dockum clevosaur [50]) and the non-monophyly of "*Clevosaurus*" resulted in most of these occurrences being accommodated in a large polytomy. Other, highly uncertain, occurrences of clevosaur-grade sphenodontians e.g. [51,52] were not included.

Archosauromorpha – Interrelations of basal archosauromorph clades follow [28,53,54], although uncertainty in the position of *Prolacertoides* limits resolution in this region. Tanystropheid phylogeny follows [55], with additional taxa added to this scaffold after [56]. Allokotosaurian internal relationships are after [53,57]; rhynchosaurian topology primarily follows [53,58] with additional information from [59–61]. Non-archosaur archosauriform relationships then also follow [53], with additional reference to [62] and *Osmolskina* positioned as a euparkeriid after [63]. Proterosuchid taxonomy and referral of specimens follows [53,64].

Pseudosuchia – Phytosauria is included within Pseudosuchia after [53]. Phytosaur taxonomy primarily follows the results of the species/specimen-level analyses of [50,65] and the summary of [66]. *Rutiodon* spp. other than *R. carolinensis* are not considered valid [66]; the wastebin nature of the taxon also means that *R. sp.* records could not be accommodated. *Pseudopalatus*, *Arribasuchus*, and *Redondasaurus* were considered junior synonyms of *Machaeroprosopus* after [67]. Phytosaur phylogeny primarily follows [68,69] with *Pravusuchus* considered the sister-taxon to Pseudopalatinae after [65]. "*Paleorhinus*" *parvus* is positioned as an *Angistorhinus*-grade phytosaur following the comparisons of [69]. The holotype of "*Angistorhinopsis*" has been generally compared to *Nicrosaurus* e.g [70] and is hence placed close to *Nicrosaurus* as a possible synonym.

Ornithosuchidae is placed after [53,71]; relationships within the clade then follow [72]. The position of the Erpetosuchidae was highly labile in the results of [73], primarily due to the taxa *Gracilisuchus* and *Turfanosuchus*, which have since been reappraised [71]. Consequently,

erpetosuchids are here placed according to their position upon removal of these taxa in [73], as sister to [*Revueltosaurus*+Aetosauria]. Aetosaur taxonomy follows [74–76] and phylogeny primarily [76] with additional input from [77]. The problematic taxa *Acompsosaurus* and *Ebrachosaurus* were excluded.

Gracilisuchid phylogeny follows [71]. Poposaurid phylogeny then follows [78]. "Prestosuchid" and rauisuchian topology follows [78–81] with *Youngosuchus* placed after [53]. However, conflict between competing topologies and differences in taxon sampling mean that resolution is this region of the tree is compromised, with "prestosuchids" forming a large polytomy. *Dagasuchus* and *Decuriasuchus* are included in this polytomy based upon the comparisons of [82,83].

Crocodylomorph taxonomy follows the summary of [84] and relationships follow [78,85–88], although incomplete taxon coverage and topological conflict means that the resolution of "sphenosuchian" taxa is poor. Although the material originally described as "*Dianchungosaurus*" is chimeric, it does demonstrate some mesoeucrocodilian synapomorphies [89] and so is retained accordingly here.

Avemetatarsalia – Avemetatarsalian relationships primarily follow [78], with additional information from [79,90–92]; uncertainty in the position of *Scleromochlus* means that it was placed in a polytomy with Pterosauria and more derived taxa. Taxonomy of basal forms follows [93], with the "Eagle Basin lagerpetid" assigned to *Dromomeron romeri* after [94]. Pterosaur taxonomy and phylogeny follows [95] and comparisons in [96]. *Faxinalipterus* is considered Archosauria indet. following [97] and so was excluded from consideration.

The "Eagle Basin silesaurid" is retained as a separate OTU following [94]. The Otis Chalk and Petrified Forest silesaurids each also preserve putative apomorphies [98] and so are also retained. *Pseudolagosuchus* was considered a junior synonym of *Lewisuchus* after [92,98,99] and *Agnosphitys* was included as a silesaurid after [93]. Silesaurid phylogeny follows [92,99–101],

although accommodation of poorly-known taxa and specimens means that resolution within this clade is poor.

Nyasasaurus was placed in a polytomy with Ornithischia and Saurischia in order to accommodate the uncertainty in its phylogenetic position [81]. Ornithischian relationships then follow [102–104] with heterodontosaurid taxonomy and phylogeny after [102,103,105,106], although accommodation of poorly-known specimens and taxa results in very limited resolution within this clade. Dubious Triassic and Early Jurassic ornithischians based on fragmentary or dental remains have been omitted [107,108]. Although the thyreophorans *Tatisaurus* and *Bienosaurus* are of dubious validity [109] they can be distinguished from *Scelidosaurus* [109] and so are provisionally retained here as informal taxa.

Theropod relationships follow [110–113]. Coelophysoid relationships follow [114], although the complicated taxonomic history of *Coelophysis* and proximate taxa [115] means that *Coelophysis bauri*, *Coeolophysis* (= *Megapnosaurus*) *rhodesiensis*, and *Campsosaurus* have been collapsed into a polytomy. Additional theropod taxa were placed on the basis of phylogenetic results or comparisons from individual studies (*Gojirasaurus* [116], *Lophostropheus* [117], *Dracovenator* [118], dilophosaurid remains from the Dharmaram Formation [119], and an unnamed neotheropod from Poland [120]). *Eshanosaurus* is tentatively considered to represent the oldest coelurosaur after [121].

Sauropodomorph relationships follow [122–136]. *Asylosaurus* was positioned in a polytomy with *Thecodontosaurus* and *Pantydraco* based upon the comparisons of [137]. *Euskelosaurus* was considered invalid after [138,139], with most material reassigned to *Plateosauravus* following [138–143]. *Plateosaurus longiceps* is treated as a junior synonym of *P. erlenbergiensis* [144]; *P. gracilis* is tentatively treated as a valid taxon following [145]. Purported *Plateosaurus* sp. records from the UK cannot be reliably diagnosed above the level of Saurischia [146].

Doubts about the validity of "*Gyposaurus sinensis*" means that most specimens formerly referred to this taxon were omitted from analysis. However, the two specimens for which the taxon were originally named are potentially valid and have been resolved in a basal position proximate to *Sarahsaurus* [147]. *Lufengosaurus magnus* was considered a likely junior synonym of *L. huenei* and *Yunnanosaurus robustus* a junior synonym of *Y. huangi* [141].

The holotype of "*Gryponyx africanus*" was considered potentially valid and placed in the position resolved by [136]. *Xixiposaurus* was positioned proximate to *Mussaurus* following the results of [148]. *Coloradisaurus* was also positioned close to these taxa in [148]. However, this is due to features which have since been demonstrated to be erroneous [124,125] and *Coloradisaurus* is instead considered a massospondylid after [124–126,131,133]. *Chinshakiangosaurus* was positioned on the basis of [149].

Synapsida

"Pelycosaur" phylogeny follows [150]. *Raranimus* is considered the sister taxon to all other therapsids after [151]. Biarmousuchian relationships follow [152,153]; *Biarmosuchoides* is tentatively positioned as a basal biarmousuchian, in a polytomy with *Biarmosuchus* and more derived taxa based on the comparisons of [154]. *Ivantosaurus* and *Eotitanosuchus* are considered junior synonyms of *Biarmosuchus tener* following [155]. Anteosaur taxonomy follows [156] and tapinocephalid taxonomy [157,158]. Dinocephalian phylogeny follows [159], although lack of recent interest in tapinocephalid interrelationships means that resolution within this clade is poor. The affinities of *Niaftasuchus* and *Phtinosuchus* within therapsids are unknown [156]; consequently, they were not included.

Anomodont phylogeny follows [160,161] with emydopoid topology following [162]. Additional general input on taxonomy comes from [163,164]. *Endothiodon* taxonomy follows [165] in the absence of formal revision, as in [164]. Relationships within the genus follow [166], with *E. tolani*

placed as the sister taxon to all other species on the basis of the comparisons of [167]. *Chelydontops* is considered a junior synonym of *Brachyprosopus* after [161] and *Diictodon tienshanensis* a junior synonym of *D. feliceps* after [168]. *Abajudon* was excluded as its affinities are poorly constrained [169]. *Kingoria* was considered synonymous with *Dicynodontoides* after [170]. Taxonomy of *Dicynodon* spp. and taxa formerly considered to belong to this genus follow [160,162,171]; the polyphyletic nature of the classical "*Dicynodon*" means that occurrences only identified to the level of *D.* sp. were omitted. "*Aulacephalodon peavoti*" has been referred to multiple species and genera, and in addition the holotype is currently lost [172]. Consequentially, it was also omitted from the dataset.

Lystrosaurus spp. taxonomy follows [164,173], with OUMNH TSK 2 removed from the genus after [171]. The affinities of "*Kannemeyeria latirostris*" are contested [174]: given that it provides redundant biogeographic information with respect to *K. lophorhinus* it was omitted from analysis. *Rechnisaurus* was considered endemic to India, with African occurrences instead referred to *Kannemeyeria* after [164,175]. *Placerias gigas* was considered synonymous with *P. hesternus* [176]. The phylogenetic position of *Elephantosaurus* is uncertain [160] and it provides redundant biogeographic information with regards to better-constrained kannemeyeriiform taxa. As a result, it was omitted here. *Sungeodon* was placed after [177].

A humerus from the Upper Muschelkalk originally compared to *Placerias* [178] has since been compared to multiple other kannemeyeriiform taxa [179,180]. Unfortunately, the specimen is now destroyed, making assessment of it even more problematical. Given these difficulties, it was omitted from analysis.

Taxonomy and phylogeny of basal gorgonopsids follow [181] and those of rubidgeines follow [182]. The holotype of "*Gorgonops whaitsi*" is both problematic and apparently lost [181]. Consequently, it was excluded. *Arctognathus curvimola* was considered the only species of

Arctognathus following [183]. *Arctops* is tentatively placed in a position close to *Smilesaurus* based on the comparisons of [182].

Viatkogorgon, *Suchogorgon*, and *Pravoslavlevia* are problematic, having never been included in a numerical phylogenetic analysis. *Kamagorgon* and *Dinosaurus* are also poorly phylogenetically constrained, having been compared variously with gorgonopsians [184], *Phtinosuchus* and dinocephalians [185]. Consequently, all of these taxa were omitted.

Therocephalian relationships primarily follow [186,187]. Taxonomy generally follows [163] with updates from subsequent phylogenetic analyses and descriptions [187–193]. The traditional "Scalposauridae" is polyphyletic [189]: as a result, scaloposaurid taxa which have yet to be included in quantitative phylogenetic analyses cannot be placed with any precision and so were excluded. *Megawhaitsia* is placed as a whaitsid after [194].

Non-mammaliaform cynodont topology primarily follows [195], with additional referral to [14,196–200] for traversodontids and [201–203] for prozostrodontians. Cynodont taxa known only from dental remains e.g. [204,205] have proven to be phylogenetically problematic [205] and so were omitted from analysis here. *Cyrbasiodon* is considered a junior synonym of *Procynosuchus* after [206]. *Procynosuchus rubidgei* is considered synonymous with *P. delharpae* after [163]. *Trirachodon* species taxonomy follows [163,207]. *Bolotriodon* was positioned after [208]; *Cromptodon* was placed proximate to *Bolotriodon* and Galesauridae based upon the comparisons of [209]. *Titanogomphodon* is considered a diademodontid following [210].

"Pachygenelus milleri" is undiagnostic above the level of ?Eucynodontia indet. [50] and so was excluded, as was the problematic "madysaurid" *Madysaurus*. *Probelesodon* and *Belesodon* were treated as junior synonyms of *Chinquodon* after [211]. *Abelobasileus* was placed following [212] and *Dinnebitrodon* was considered to be a tritylodont after [213]. "Dromatheriids" are poorly known, and their phylogenetic position remains uncertain [214,215], preventing their inclusion.

The same is true for most “therioherpetids”, which have not been included in quantitative phylogenetic analyses. The exceptions to this are *Therioherpeton* and *Riograndia*, which have generally not been found to form a monophyletic group [195]. Other “therioherpetids” hence could not be accommodated. *Oligokyphus* spp. referral follows [216]. *Protheriodon, Panchetocynodon*, and *Minicynodon* are positioned based on the results and comparisons of [209].

The interrelationships of basal mammaliaform clades follow [199,217–222]. *Hadrocodium* was positioned after [223] and *Trishulotherium* after [224]. *Dyskritodon* is considered a triconodontid [225] and *Brachyzostrodon* a megazostrodontid with the informal species of [226] provisionally included. The content of the Morganucodontidae follows [227,228] and that of Haramiyidae follows [199,229]. *Indobaatar* is considered to represent the earliest multituberculate after [230]. Mammaliaform tooth taxa (e.g. “symmetrodonts”) are generally phylogenetically and taxonomically problematic, and so were mostly excluded, as with non-mammaliaform cynodonts.

Supplementary Note 2: Taxon occurrences and ages

Occurrence data for these taxa was taken primarily from the Paleobiology Database [231], with the addition of some occurrences from the literature (see Supplementary Data 2). Taxa were dated at stage level. They were then placed in the following time bins for analysis: Lopingian, Early Triassic (Induan and Olenekian), Anisian, Ladinian, early Late Triassic, late Late Triassic, early Early Jurassic (Hettangian, Sinemurian), and late Early Triassic (Pliensbachian, Toarcian).

The Late Triassic was not split into its constituent stages due to the disproportionately long Norian e.g. [232–235]. Similarly, recent re-dating of the Los Colorados Formation as being early–middle Norian in age [235] prevents separation of time bins according to the Ischigualastian and

Coloradian land vertebrate ages. Instead, Late Triassic occurrences were divided into two time bins: Carnian–early Norian (Lacian) and middle/late Norian (Alaunian)–Rhaetian, with the separation between these bins dated at approximately 219–213 Ma [232]. Geological units were assigned to each of these time bins as summarized in Supplementary Tables 1 and 2. However, difficulties in the dating of individual geological units and/or uncertainty in dating the base of the Alaunian [232] meant that occurrences from the Sonsela Member of the Chinle Formation, the Trujillo Formation, the Lockatong and lower Passaic (Neshanician) formations from the Newark Basin, the Leedstown Formation from the Taylorville Basin, Late Triassic occurrences from the Fundy Basin, the Bigoudine Formation, the *Riograndia* Assemblage Zone of the Santa Maria Supersequence, the La Esquina fauna of the Los Colorados Formation, the Upper Maleri Formation, the fissure-fill deposits of the southwestern UK, and most Middle Stubensandstein occurrences could not be satisfactorily restricted to a single time bin. To reflect this uncertainty, these occurrences were included in both Late Triassic time bins for analysis.

Area	early Late Triassic	Referral
**Western USA**	Chinle Group: Mesa Redondo Member, Bluewater Creek Formation, Blue Mesa Member.<br><br>Dockum Group: Colorado City Formation, Tecovas Formation and correlates of these units.	Otischalkian and Adamanian Land Vertebrate Faunas [236] were considered to be Carnian and early Norian in age, respectively, after [233]. This corresponds with radiometric dating of the overlying Sonsela Formation of the Chinle Group [237].
**Eastern USA**	Newark Supergroup: Stockton Formation, Conewagian Formation, New Oxford Formation, Cumnock Formation.<br><br>Taylorsville Basin: Newfound Formation, Port Royal Formation.	The chronostratigraphic framework of the Newark Supergroup and its correlates used herein follows that of [234].
**Morroco**	Irohalene Formation	The Irohalene Formation is dated late Carnian-early Norian on the basis of biostratigraphy [238] and palynological dating of the overlying Bigoudine Formation [239,240].
**UK**	Lossiemouth Sandstone	The Lossiemouth Sandstone is considered to be late Carnian in age on the basis of biostratigraphy [91].
**Continental Europe**	Germanic Basin: Stuttgart Formation, Weser Formation, Arnstadt Formation, Lower Stubensandstein of the Lowenstein Formation and their correlates.<br><br>Poland: the Krasiejow fauna.	The Lacian is considered to be early Norian in age, following [232,241–244]. The Krasiejow fauna is dated after [245–247].
**Brazil**	Santa Maria Supersequence: Santa Cruz Sequence, Candelaria Sequence.	Stratigraphy and dating of the Santa Maria Supersequence follows [248], with these sequences being considered to range from late Ladinian to early Norian.
**Argentina**	Ischigualasto Formation, Cacheuta Formation, the La Chilca Fauna of the Los Colorados Formation.	Stratigraphy follows [249], biostratigraphy [100,127] and chronostratography [235]. The Cacheuta Formation is dated based on correlation with the Ischigualasto Formation [240,250]. The La Chilca fauna is correlated with the Candelaria Sequence based on the presence of *Jachaleria* [240].
**India**	Lower Maleri Formation	The Lower Maleri fauna allows correlation with the *Scaphonyx-Exaeretodon-Herrerasaurus* and *Hyperodapedon* Assemblage Zones, and so is late Carnian to early Norian in age [132].

Supplementary Table 1: Referral of stratigraphic units to the early Late Triassic time bin as used in this analysis. For units referred to the late Late Triassic time bin see Supplementary Table 2; for those which could not be delimited to either Late Triassic time bin, and so were included in both, see Supplementary Note 2.

Area	late Late Triassic	Referral
**Western USA**	Chinle Group: upper Petrified Forest/Painted Desert Member, Owl Rock Member, Rock Point Member.<br><br>Dockum Group: Redonda Formation, Sloan Canyon Formation, Upper Trassever Formation, and correlates of these units.	The Apachean Land Vertebrate Fauna [236] was considered to be late Norian-Rhaetian in age, after [233].
**Eastern USA**	Newark Supergroup: Upper Paissic Formation (Cliftonian).<br><br>New Haven Formation.	Newark Supergroup chronostratigraphy follows [234]. The age of the New Haven Formation is based on the radiometric dates of [251].
**Greenland**	Fleming Ford Formation	The Fleming Ford Formation is considered Rhaetian based on correlation with the Newark-APTS 2010 [234] and the Steinbergkogel [252].
**Continental Europe**	Germanic Basin: Upper Stubensandstein of the Lowenstein Formation, Trossingen Formation (Knollenmergel), Exter Formation, and their correlates.<br><br>Italy: Zorzino Limestone, Dolomia di Forni Formation.	The Sevatian 1 is considered to be late Norian in age, and Sevatian 2 Rhaetian in age following [232,241–244]. The Italian limestone successions are dated on the basis of palynofloras and conodont ages [253–255].
**Argentina**	Quebrada del Barro Formation	[256].
**South Africa**	Lower Elliot Formation	[257].
**India**	Dharmaram Formation	The absence of rhynchosaurs and therapsids in this fauna imply a late Norian-Rhaetian age [132].

Supplementary Table 2: Referral of stratigraphic units to the late Late Triassic time bin as used in this analysis. For units referred to the early Late Triassic time bin see Supplementary Table 1; for those which could not be delimited to either Late Triassic time bin, and so were included in both, see Supplementary Note 2.

Supplementary Note 3: Geographic regions

Geographic areas for use in studies of palaeobiogeography are usually defined on the basis of modern continental configurations e.g. [258–261]. However, this is potentially problematic when dealing with occurrences on a supercontinent where, for example, eastern North American and northwestern African localities were much closer to each other than to localities in southwestern North America or southern Africa, respectively. Instead, the geographic areas to be treated as nodes in the network analyses were defined on the basis of multivariate *k*-means clustering of palaeocoordinate data.

Palaeolongitudes and palaeolatitudes for 2144 Lopingian-Toarcian terrestrial tetrapod occurrences were taken from the Paleobiology Database, and binned at epoch level, so as to mitigate the effects of continental drift on results (see Supplementary Data 2). *K*-means clustering was then performed on these epoch-level datasets within R [3], varying *k* from 5-15. Ten-thousand replicates were performed for each of these analyses, with ten random starts. The performance of each was measured as the proportion of the total variance explained by the resolved clusters (the ratio of the between clusters sum of squares: total sum of squares). The best performing iteration for each value of *k* was retained for further comparison.

Comparison between results of different values of *k* was principally performed on the basis of the proportion of variance explained by each, with those scoring <98% omitted from consideration. These results are given in Supplementary Table 3, with full results of the cluster analyses given as Supplementary Data 3. Further comparison between results for different values of *k* was performed on the following criteria: the consistency of the clusters through the time interval of interest and their consistency with previously recognized biogeographic provinces e.g. [240,260,262]. Results for fifteen clusters were unstable between different time bins, resulting in the designation of ten clusters to use in the network biogeography analyses, as

given in Fig. 1b. These then formed the basis of the bipartite taxon-locality presence/absence matrices for each time bin, given in Supplementary Data 4.

	Number of clusters (*k*)										
	**5**	**6**	**7**	**8**	**9**	**10**	**11**	**12**	**13**	**14**	**15**
**late Permian**	97.3	97	97.3	97.4	98.1	98.7	98.8	98.8	98.9	99	99.1
**Early Triassic**	93.7	96.5	97.5	98	97.8	98.6	98.8	99.2	99.4	99.5	99.5
**Middle Triassic**	93	95.8	96.9	97.3	98.1	98.2	98.9	98.8	99.3	99.3	99.4
**Late Triassic**	91.8	93.6	94.8	95.6	96.9	98	97.6	97.9	97.7	97.9	98.7
**Early Jurassic**	97.6	98.5	99.2	99.5	99.5	99.6	99.6	99.6	99.7	99.8	99.8

Supplementary Table 3: Performance values (measured as the % total variance explained by the observed clusters) of the best performing clustering analyses for each value of *k* in each time bin. Those exceeding the 98% threshold applied within are shaded.

Supplementary Note 4: Sampling

Global sampling during this interval is uneven: in particular, there is a dearth of occurrences from low-latitude Gondwana during the late Permian, Ladinian, latest Triassic, and Early Jurassic (Supplementary Fig. 1). During the Early Triassic, at least, this paucity may have been genuine [264], but may be masking biogeographical heterogeneity in other bins [265]. Still, the inclusion of phylogenetic information, and use of relatively broad regions, makes the analyses reported herein less vulnerable to sampling heterogeneity than similar previous studies.

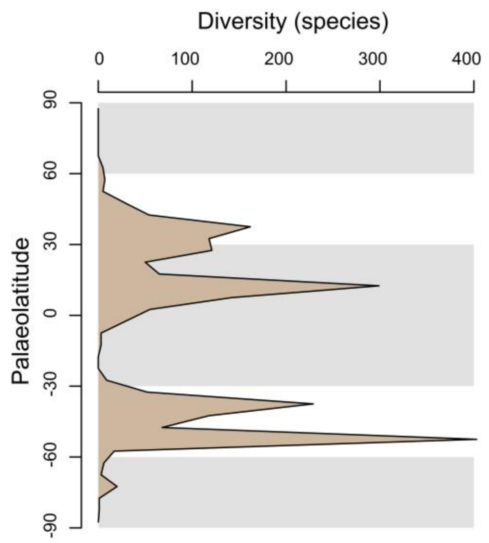

Supplementary Figure 1: Plot of the number of late Permian-Early Jurassic species sampled in this analysis against palaeolatitude.

Global pBC shows no significant correlation with either the number of taxa or the number of regions (and so, localities) sampled in each time bin (Supplementary Figs 2, 3a, b). In contrast, a significant negative correlation is observed between global pBC and the length of bins as used in the main results (Supplementary Fig. 3c). This suggests that the low values of pBC observed in the Late Triassic and Early Jurassic may be a consequence of the relative length of these bins.

To investigate this further, the analysis was repeated with shorter-scale time bins within this interval: Carnian-early Norian, late Norian, Rhaetian, Hettangian, Sinemurian, Pliensbachian, and Toarcian (unfortunately difficulty separating late Carnian and early Norian occurrences prevented subdivision of the early Late Triassic). The same trends are resolved using these shorter time bins: low pBC during the Late Triassic, followed by a significant increase across the Triassic-Jurassic boundary after which values decline strongly in the Pliensbachian-Toarcian (Supplementary Fig. 4). Under this treatment, the relationship between pBC and bin length is no longer significant (Supplementary Fig. 3d): the late Norian and Rhaetian represent two of the shortest time bins, yet still exhibit pBC values of close to zero. Overall results

therefore appear robust to binning treatment and are not driven by bin length. As a result, the analyses herein are considered to capture valid biological signal, especially in Laurasia and temperate Gondwana.

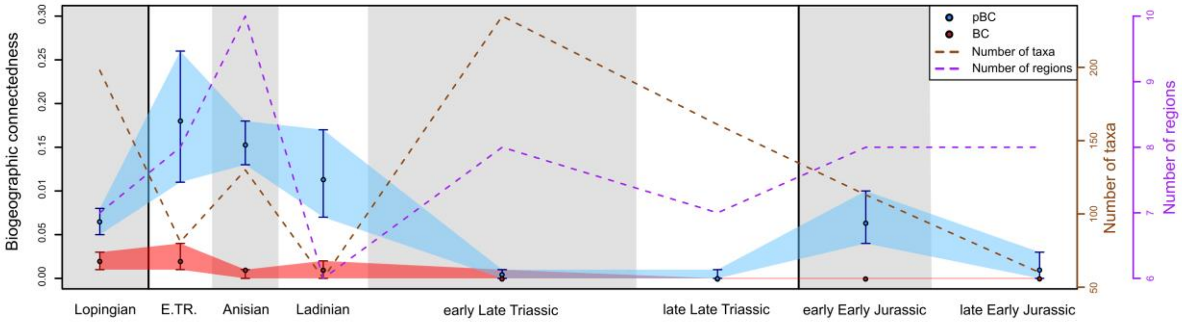

Supplementary Figure 2: Results for phylogenetic biogeographic connectedness (pBC, in blue, 95% confidence intervals calculated from jackknifing with 10,000 replicates), non-phylogenetic biogeographic connectedness (BC, in red, 95% confidence intervals calculated from jackknifing with 10,000 replicates) for the Lopingian through to the end of the Early Jurassic, plotted against the number of species and number of regions in each time bin.

However, the poor and uneven sampling of low-latitude Gondwanan localities means that results for the southern hemisphere should still be interpreted more cautiously. This is particularly true in the late Late Triassic time bin, which contains only 29 Gondwanan species, of which over 48% are known from a single area. Consequently, the potential decoupling of local pBC signals within Gondwana from global patterns identified in this time bin requires corroboration from future sampling of additional Gondwanan localities.

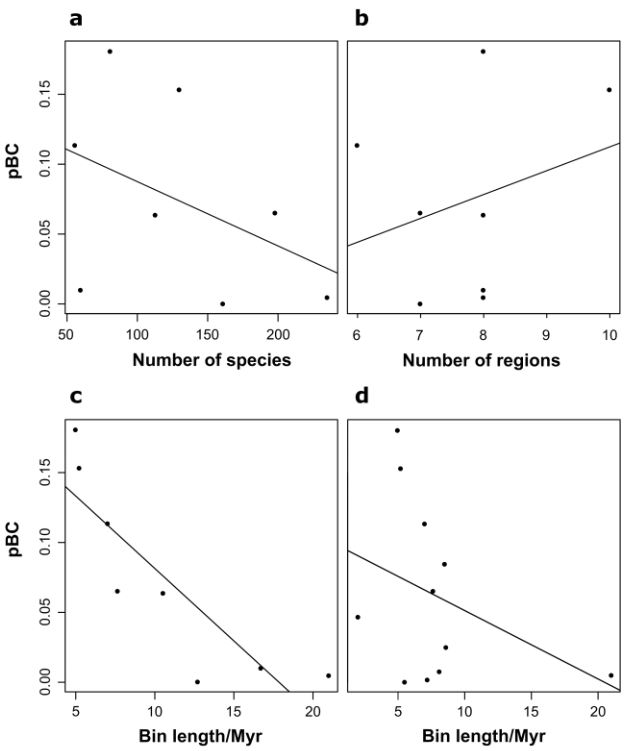

Supplementary Figure 3: Sampling of taxon occurrence data. a) Linear regression of phylogenetic biogeographic connectedness versus the number of species included in each time bin, showing a non-significant relationship (p = 0.2855, adjusted r^2 = 0.05076). b) Linear regression of phylogenetic biogeographic connectedness versus the number of regions in each time bin, showing a non-significant relationship (p = 0.4924, adjusted r^2 = -0.07128). c) Linear regression of phylogenetic biogeographic connectedness versus the length of each time bin, showing a significant relationship (p = 0.0060103, adjusted r^2 = 0.6971). d) Linear regression of pBC against time bin length when subdividing the Late Triassic and Early Jurassic into shorter time bines (see Supplementary Note 4, Supplementary Fig. 5), showing a non-significant relationship (p = 0.2648, adjusted r^2 = 0.03979).

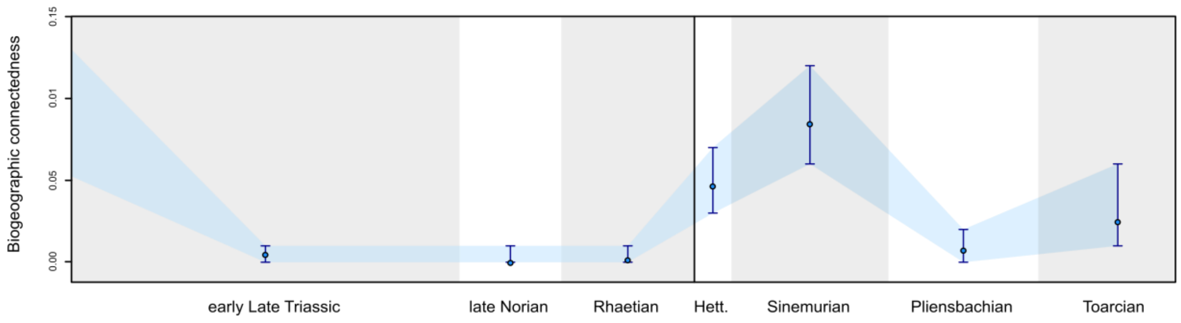

Supplementary Figure 4: Phylogenetic biogeographic connectedness results for the Late Triassic-Early Jurassic interval, employing finer time bins for the late Late Triassic and Early Jurassic. Ninety-five percent confidence intervals, calculated from jackknifing with 10,000 replicates, are given. Hett. = Hettangian.

Supplementary Note 5: Clade age sensitivity analysis

Analysis of taxonomic subsets demonstrated that the significant pBC increases observed across both the Permian-Triassic and Triassic-Jurassic boundaries were primarily driven by the radiation of novel taxa. An alternative possibility, however, is that this increase in pBC is an artefact related to a lower average clade age within these time bins. To investigate this possibility, the analysis was repeated for the entire time interval including only those clades which diverged within 2Ma above or below the lower boundary of each time bin. Unfortunately, resulting very low sample sizes in the Ladinian and latest Triassic necessitated the use of epoch-level bins for this analysis.

The results of this analysis recapitulate the overall signal seen from analysis of the complete dataset (Supplementary Fig. 5). Significant increases in pBC are still observed across both mass extinction boundaries, and there is no significant difference between pBC values derived from this subset or the total dataset in Triassic time bins. This indicates that the primary biogeographic signals observed during this interval – a decline in cosmopolitanism through the sampled interval, punctuated by increases across both the Permian-Triassic and Triassic-Jurassic boundaries – is not merely an artefact resulting from the average clade age within each time bin.

In the Lopingian time bin the pBC of these youngest clades was significantly greater than from all clades, a pattern otherwise only seen in the post-extinction time bins. The Lopingian is predated the poorly-understood end Guadalupian event, which may represent another mass extinction[266]. This offset may reflect similar biogeographic patterns accompanying turnover at this time: further analysis of a larger late Palaeozoic interval will be required to test this further.

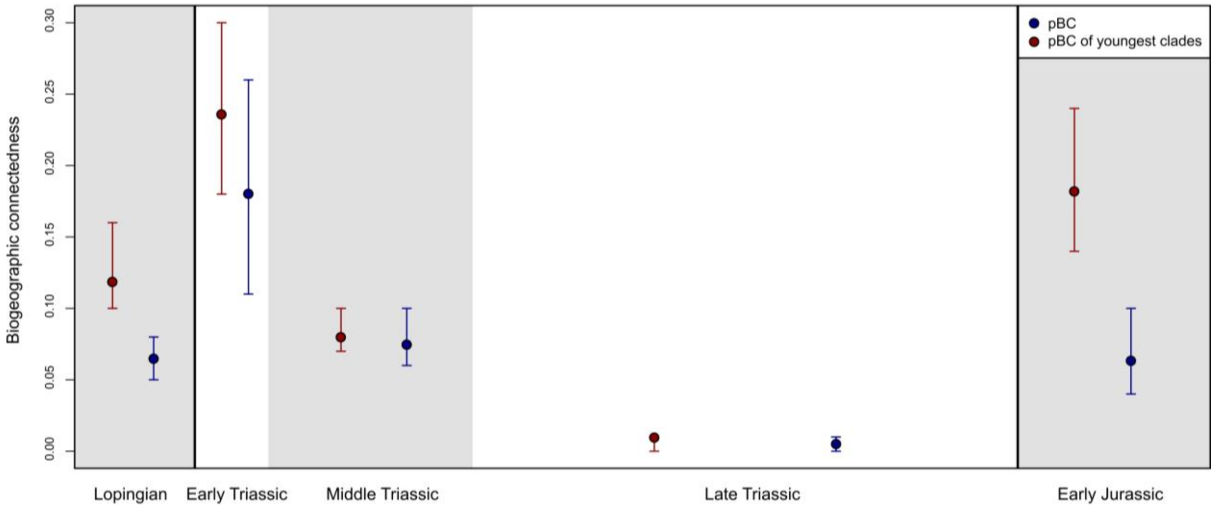

Supplementary Figure 5: Phylogenetic biogeographic connectedness results through the Lopingian-Early Jurassic, comparing results from the whole dataset (in blue) with those from a subset including only clades diverging within 2Ma either side of the lower boundary of each bin (in red). Ninety-five percent confidence intervals, calculated from jackknifing with 10,000 replicates, are given.

Supplementary Note 6: Phylogenetic distances in each time bin

It should be noted that a given value of pBC does not represent a unique solution, as the same value could be theoretically generated by a few links between closely-related taxa or more links between more moderately related taxa.

To ensure that observed pBC results are not being driven purely by differences in the average branch lengths between taxa sampled in each time bin, pairwise distances, based on branch lengths, between all tips were calculated across all input trees for each time bin, following the truncation of maximum branch lengths to the μ-value (see Supplementary Note 7). Results

indicate no significant differences between time bins (Supplementary Fig. 6), confirming that pBC results are not redundant with respect to average relatedness of sampled taxa.

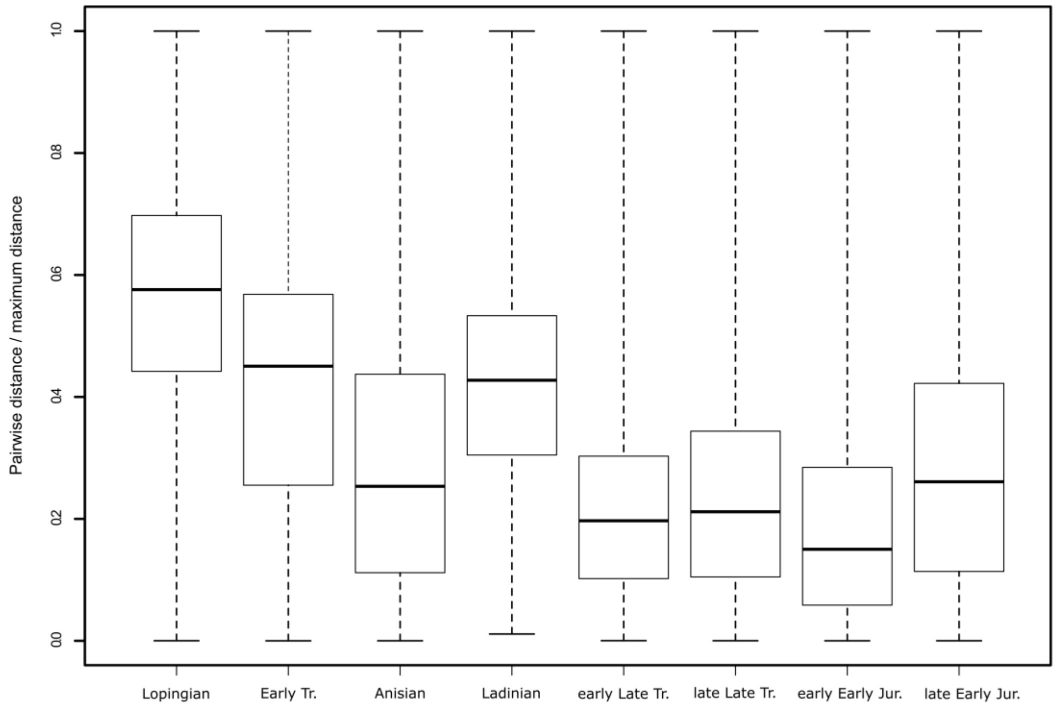

Supplementary Figure 6: Descriptive statistics of pairwise distances between all tips as measured across all 100 input trees for each time bin after truncation by a μ value of 15Ma, scaled against the maximum distance between two tips in each bin. Solid lines refer to median values, box margins to the interquartile range, and whiskers to the maximum and minimum observed values in each bin. Tr. = Triassic, Jur. = Jurassic.

Supplementary Note 7: Phylogenetic network biogeography μ sensitivity analyses

Analysis of a simulated null (stochastically generated) dataset indicated a systematic bias towards increasing phylogenetic biogeographic connectedness (pBC) through time. This is due to the increasing distance from a persistent root to the tips through time, which results in phylogenetic branch lengths between nearest relative terminal taxa becoming proportionately shorter. This was mitigated through the introduction of a constant, μ, which collapses all branches below a fixed "depth" such that root age is equal to μ million years from the tips.

The introduction of this constant also alleviates problems of temporal superimposition of biogeographic signals which may otherwise occur. It means that pBC results reported for each time bin reflect patterns generated by biogeographic processes in the preceding μ million years, preventing these recent biogeographic signals of interested from being swamped by those from deeper time intervals.

Sensitivity analyses varying the value of μ from 1–25 were performed on the Lopingian-Toarcian taxon-region matrices (Supplementary Fig. 7). The results were used to choose a μ value of 15 for further analysis, for which final results are presented in the main text. Note that changes in the value of μ make no difference to the relative pBC seen in consecutive time bins, and so the increases observed across both the Permian-Triassic and Triassic-Jurassic mass extinction boundaries are not sensitive to different values of μ. A decrease from high values of pBC in the Middle Triassic to very low values in the Late Triassic is also robust to the value of μ chosen.

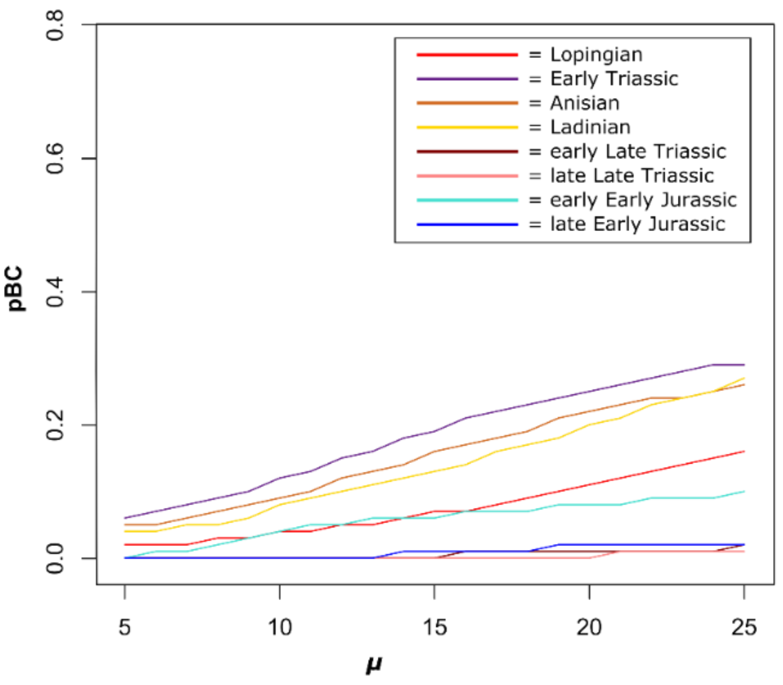

Supplementary Figure 7: Results of sensitivity analyses on varying the value of μ from 5-25 in the calculation of phylogenetic biogeographic connectedness (pBC).

Once the value of μ had been chosen for further analysis, pBC was computed for each time bin. Confidence intervals for each bin were then calculated through jackknifing of occurrence records, with 10,000 replicates. This method has been made available as the "BC" function within the R package dispeRse ([263], github.com/laurasoul/dispeRse). Additional options include bootstrapping and jackknifing of occurrence records, permutation of random trees, and measuring phylogenetic proximity by counting nodes as opposed to measuring branch lengths. An example script for pBC analyses is given in Supplementary Data 5, and a more comprehensive script covering the analyses performed in this study as Supporting Data 6.

Supplementary references

1. Goloboff, P. A. & Pol, D. Semi-strict supertrees. *Cladistics* **18,** 514–525 (2002).
2. Bapst, D. W. Paleotree: An R package for paleontological and phylogenetic analyses of evolution. *Methods Ecol. Evol.* **3,** 803–807 (2012).
3. Team, R. development core. R: A language and environment for statistical computing. *R Found. Stat. Comput. Vienna, Austria.* **ISBN 3-900,** (2008).
4. Cisneros, J. C. Phylogenetic relationships of procolophonid parareptiles with remarks on their geological record. *J. Syst. Palaeontol.* **6,** 345–366 (2008).
5. Tsuji, L. A. & Müller, J. Assembling the history of the Parareptilia: Phylogeny, diversification, and a new definition of the clade. *Foss. Rec.* **12,** 71–81 (2009).
6. Tsuji, L. A., Sobral, G. & Müller, J. *Ruhuhuaria reiszi,* a new procolophonoid reptile from the Triassic Ruhuhu Basin of Tanzania. *Comptes Rendus - Palevol* **12,** 487–494 (2013).
7. Säilä, L. K. The phylogenetic position of *Nyctiphruretus acudens,* a parareptile from the Permian of Russia. *J. Iber. Geol.* **36,** 123–143 (2010).

8. Ruta, M., Cisneros, J. C., Liebrecht, T., Tsuji, L. A. & Müller, J. Amniotes through major biological crises: Faunal turnover among Parareptiles and the end-Permian mass extinction. *Palaeontology* **54,** 1117–1137 (2011).

9. Macdougall, M. J. & Reisz, R. A new parareptile (Parareptilia, Lanthanosuchoidea) from the Early Permian of Oklahoma. *J. Vertebr. Paleontol.* **32,** 1018–1026 (2012).

10. Reisz, R. R., Macdougall, M. J. & Modesto, S. P. A new species of the parareptile genus *Delorhynchus*, based on articulated skeletal remains from Richards Spur, Lower Permian of Oklahoma. *J. Vertebr. Paleontol.* **34,** 1033–1043 (2014).

11. Modesto, S. P. *et al.* The oldest parareptile and the early diversification of reptiles. *Proc. Biol. Sci.* **282,** 20141912 (2015).

12. Tsuji, L. A., Müller, J. & Reisz, R. R. Anatomy of *Emeroleter levis* and the phylogeny of the nycteroleter parareptiles. *J. Vertebr. Paleontol.* **32,** 45–67 (2012).

13. Tsuji, L. A. Anatomy, cranial ontogeny and phylogenetic relationships of the pareiasaur *Deltavjatia rossicus* from the Late Permian of central Russia. *Earth Environ. Sci. Trans. R. Soc. Edinburgh* **104,** 81–122 (2013).

14. Xu, L., Li, X.-W., Jia, S.-H. & Liu, J. The Jiyuan tetrapod Fauna of the Upper Permian of China--1. New pareiasaur material and the reestablishment of *Honania complicidentata*. *Acta Palaeontol. Pol.* **60**, 689-700 (2015).

15. Benton, M. J. The Chinese pareiasaurs. *Zool. J. Linn. Soc.* **177**, 813-853 (2016).

16. Jalil, N. E. & Janvier, P. Les pareiasaures (Amniota, Parareptilia) du Permien supérieur du Bassin d'Argana, Maroc. *Geodiversitas* **27,** 35–132 (2005).

17. Macdougall, M. J. & Modesto, S. P. New information on the skull of the Early Triassic parareptile *Sauropareion anoplus*, with a discussion of tooth attachment and

replacement in procolophonids . *J. Vertebr. Paleontol.* **31,** 270–278 (2011).

18. Cisneros, J. C. & Ruta, M. Morphological diversity and biogeography of procolophonids (Amniota: Parareptilia). *J. Syst. Palaeontol.* **8,** 607–625 (2010).

19. Säilä, L. K. Osteology of *Leptopleuron lacertinum* Owen, a procolophonoid parareptile from the Upper Triassic of Scotland, with remarks on ontogeny, ecology and affinities. *Earth Environ. Sci. Trans. R. Soc. Edinburgh* **101,** 1–25 (2010).

20. Falconnet, J., Andriamihaja, M., Läng, É. & Steyer, J. S. First procolophonid (Reptilia, Parareptilia) from the Lower Triassic of Madagascar. *Comptes Rendus - Palevol* **11,** 357–369 (2012).

21. Evans, S. E. The Early Triassic 'lizard' *Colubrifer campi*: A reassessment. *Palaeontology* **44,** 1033–1041 (2001).

22. Spencer, P. S. & Benton, M. J. in *The age of dinosaurs in Russia and Mongolia* (eds. Benton, M. J., Shishkin, D., Unwin, D. M. & Kurochkin, E. N.) 1601–76 (Cambridge University Press, 2000).

23. Small, B. J. A new procolophonid from the Upper Triassic of Texas, with a description of tooth replacement and implantation. *J. Vertebr. Paleontol.* **17,** 674–678 (1997).

24. Sues ', H.-D., Olsen2, P. E., Scott3, D. M. & Spencer ', P. S. Cranial osteology of *Hypsognathus fenneri*, a latest Triassic procolophonid reptile from the Newark Supergroup of eastern North America. *J. Vertebr. Paleontol.* **20,** 275–284 (2000).

25. Modesto, S. P., Scott, D. M., Botha-Brink, J. & Reisz, R. R. A new and unusual procolophonid parareptile from the Lower Triassic Katberg Formation of South Africa. *J. Vertebr. Paleontol.* **30,** 37–41 (2010).

26. Borsuk-Białynicka, M. & Lubka, M. Procolophonids from the early triassic of Poland.

Palaeontol. Pol. **65,** 107–144 (2009).

27. Reisz, R. R., Modesto, S. P. & Scott, D. M. A new Early Permian reptile and its significance in early diapsid evolution. *Proc. R. Soc. B Biol. Sci.* **278,** 3731–3737 (2011).

28. Ezcurra, M. D., Scheyer, T. M. & Butler, R. J. The origin and early evolution of Sauria: Reassessing the Permian Saurian fossil record and the timing of the crocodile-lizard divergence. *PLoS One* **9,** e89165 (2014).

29. Reisz, R. R., LeBlanc, A. R. H., Sidor, C. A., Scott, D. & May, W. A new captorhinid reptile from the Lower Permian of Oklahoma showing remarkable dental and mandibular convergence with microsaurian tetrapods. *Naturwissenschaften* **102,** 50 (2015).

30. Modesto, S. P., Lamb, A. J. & Reisz, R. R. The captorhinid reptile *Captorhinikos valensis* from the lower Permian Vale Formation of Texas, and the evolution of herbivory in eureptiles. *J. Vertebr. Paleontol.* **34,** 291–302 (2014).

31. Senter, P. Phylogeny of Drepanosauridae (Reptilia: Diapsida). *J. Syst. Palaeontol.* **2,** 257–268 (2004).

32. S. Renesto, J. A. Spielmann, S. G. Lucas, G. T. Spagnoli, The taxonomy and paleobiology of the Late Triassic (Carnian-Norian: Adamanian-Apachean) drepanosaurs (Diapsida: Archosauromorpha: Drepanosauromorpha). *New Mex. Museum Nat. Hist. Sci. Bull.* **46**, 1–81 (2010).

33. Alifanov, V. R. & Kurochkin, E. N. *Kyrgyzsaurus bukhanchenko*i gen. et sp. nov., a new reptile from the triassic of southwestern Kyrgyzstan. *Paleontol. J.* **45,** 639–647 (2011).

34. Gauthier, J., Estes, R. & de Queiroz, K. in *Phylogenetic relationships of the lizard families* (eds. Estes, R. & Pregill, G.) 303–321 (Stanford University Press, 1988).

35. Evans, S. E. & Borsuk-Białynicka, M. A small lepidosauromorph reptile from the early Triassic of Poland. *Palaeontol. Pol.* **65,** 179–202 (2009).

36. Evans, S. E. & Jones, M. E. H. i*Lecture Notes in Earth Sciences* **132,** 27–71 (2010).

37. Evans, S. E. An early kuehneosaurid reptile from the early Triassic of Poland. *Palaeontol. Pol.* **65,** 145–178 (2009).

38. Jones, M. E. H. *et al.* Integration of molecules and new fossils supports a Triassic origin for Lepidosauria (lizards, snakes, and tuatara). *BMC Evol. Biol.* **13,** 208 (2013).

39. Renesto, S. & Bernardi, M. Redescription and phylogenetic relationships of *Megachirella wachtleri* Renesto et Posenato, 2003 (Reptilia, Diapsida). *Palaontologische Zeitschrift* **88,** 197–210 (2014).

40. Hutchinson, M. N., Skinner, a. & Lee, M. S. Y. *Tikiguania* and the antiquity of squamate reptiles (lizards and snakes). *Biol. Lett.* **8,** 665–669 (2012).

41. Evans, S. E. At the feet of the dinosaurs: tThe early history and radiation of lizards. *Biol. Rev. Camb. Philos. Soc.* **78,** 513–551 (2003).

42. Evans, S. E., Prasad, G. V. R. & Manhas, B. K. Fossil lizards from the Jurassic Kota Formation of India. *J. Vertebr. Paleontol.* **22,** 299–312 (2002).

43. Heckert, A. B., Lucas, S. G., Rinehart, L. F. & Hunt, A. P. A new genus and species of sphenodontian from the Ghost Ranch *Coelophysis* quarry (upper Triassic: Apachean), rock point formation, New Mexico, USA. *Palaeontology* **51,** 827–845 (2008).

44. Apesteguía, S., Gómez, R. O. & Rougier, G. W. A basal sphenodontian (Lepidosauria)

from the Jurassic of Patagonia: New insights on the phylogeny and biogeography of Gondwanan rhynchocephalians. *Zool. J. Linn. Soc.* **166,** 342–360 (2012).

45. Rauhut, O. W. M., Heyng, A. M., López-Arbarello, A. & Hecker, A. A New Rhynchocephalian from the Late Jurassic of Germany with a Dentition That Is Unique amongst Tetrapods. *PLoS One* **7,** e46839 (2012).

46. Martínez, R. N. *et al.* A new sphenodontian (Lepidosauria: Rhynchocephalia) from the Late Triassic of Argentina and the early origin of the herbivore opisthodontians. *Proc. R. Soc. B Biol. Sci.* **280,** 20132057 (2013).

47. Apesteguía, S. & Carballido, J. L. A new eilenodontine (Lepidosauria, Sphenodontidae) from the Lower Cretaceous of central Patagonia. *1. J. Vertebr. Paleontol.* **34,** 303–317 (2014).

48. Fraser, N. New Triassic sphenodontids from southwest England and a review of their classification. *Palaeontology* **29,** 165–186 (1986).

49. Hsiou, A. S., De França, M. A. G. & Ferigolo, J. New data on the clevosaurus (Sphenodontia: Clevosauridae) from the upper triassic of southern Brazil. *PLoS One* **10,** e0137523 (2015).

50. J. W. Martz *et al.*, A taxonomic and biostratigraphic re-evaluation of the Post Quarry vertebrate assemblage from the Cooper Canyon Formation (Dockum Group, Upper Triassic) of southern Garza County, western Texas. *Earth Environ. Sci. Trans. R. Soc. Edinburgh* **103**, 339-364 (2013).

51. Delsate, D., Duffin, C. J. & Weis, R. A new microvertebrate fauna from the Middle Hettangian (Early Jurassic) of Fontenoille (Province of Luxembourg, south Belgium). *Mem. Geol. Surv. Belgium* **48,** 83pp (2002).

52. Heckert, A. B., Lucas, S. G. & Hunt, A. P. Triassic vertebrate fossils in Arizona. *New Mex. Museum Nat. Hist. Sci. Bull.* **29,** 16–44 (2005).

53. Ezcurra, M. D. The phylogenetic relationships of basal archosauromorphs, with an emphasis on the systematics of proterosuchian archosauriforms. *PeerJ* **4,** e1778 (2016).

54. Pinheiro, F. L., França, M. A. G., Lacerda, M. B., Butler, R. J. & Schultz, C. L. An exceptional fossil skull from South America and the origins of the archosauriform radiation. *Sci. Rep.* **6,** 22817 (2016).

55. Pritchard, A. C., Turner, A. H., Nesbitt, S. J., Irmis, R. B. & Smith, N. D. Late Triassic tanystropheids (Reptilia, Archosauromorpha) from northern New Mexico (Petrified Forest Member, Chinle Formation) and the biogeography, functional morphology, and evolution of Tanystropheidae. *J. Vertebr. Paleontol.* **35,** e911186 (2015).

56. Modesto, S. P. & Sues, H. D. The skull of the Early Triassic archosauromorph reptile *Prolacerta broomi* and its phylogenetic significance. *Zool. J. Linn. Soc.* **140,** 335–351 (2004).

57. Nesbitt, S. J. *et al.* Postcranial osteology of *Azendohsaurus madagaskarensis* (?Middle to Upper Triassic, Isalo Group, Madagascar) and its systematic position among stem archosaur reptiles. *Bull. Am. Museum Nat. Hist.* **398,** 1–126 (2015).

58. Butler, R. J., Ezcurra, M. D., Montefeltro, F. C., Samathi, A. & Sobral, G. A new species of basal rhynchosaur (Diapsida: Archosauromorpha) from the early Middle Triassic of South Africa, and the early evolution of Rhynchosauria. *Zool. J. Linn. Soc.* **174,** 571–588 (2015).

59. Montefeltro, F. C., Langer, M. C. & Schultz, C. L. Cranial anatomy of a new genus of

hyperodapedontine rhynchosaur (Diapsida, Archosauromorpha) from the Upper Triassic of southern Brazil. *Earth Environ. Sci. Trans. R. Soc. Edinburgh* **101,** 27–52 (2010).

60. Montefeltro, F. C., Bittencourt, J. S., Langer, M. C. & Schultz, C. L. Postcranial anatomy of the hyperodapedontine rhynchosaur *Teyumbaita sulcognathus* (Azevedo and Schultz, 1987) from the Late Triassic of Southern Brazil. *J. Vertebr. Paleontol.* **33,** 67–84 (2013).

61. Mukherjee, D. & Ray, S. A new *Hyperodapedon* (Archosauromorpha, Rhynchosauria) from the Upper Triassic of India: Implications for rhynchosaur phylogeny. *Palaeontology* **57,** 1241–1276 (2014).

62. Desojo, J. B., Ezcurra, M. D. & Schultz, C. L. An unusual new archosauriform from the Middle-Late Triassic of southern Brazil and the monophyly of Doswelliidae. *Zool. J. Linn. Soc.* **161,** 839–871 (2011).

63. Sookias, R. B. The relationships of the Euparkeriidae and the rise of Archosauria. *R. Soc. Open Sci.* **3,** 150674 (2016).

64. Ezcurra, M. D. & Butler, R. J. Taxonomy of the proterosuchid archosauriforms (Diapsida: Archosauromorpha) from the earliest Triassic of South Africa, and implications for the early archosauriform radiation. *Palaeontology* **58,** 141–170 (2015).

65. Stocker, M. R. A new taxon of phytosaur (Archosauria: Pseudosuchia) from the Late Triassic (Norian) Sonsela Member (Chinle Formation) in Arizona, and a critical reevaluation of *Leptosuchus* Case, 1922. *Palaeontology* **53,** 997–1022 (2010).

66. Stocker, M. R. & Butler, R. J. Phytosauria. *Geol. Soc. London, Spec. Publ. Spec. Publ.*

379, 91–117 (2013).

67. W. G. Parker, A. Hungerbühler, J. W. Martz, The taxonomic status of the phytosaurs (Archosauriformes) *Machaeroprosopus* and *Pseudopalatus* from the Late Triassic of the western United States. *Earth Environ. Sci. Trans. R. Soc. Edinburgh* **103**, 265–268 (2013).

68. Hungerbühler, A., Mueller, B., Chatterjee, S. & Cunningham, D. P. Cranial anatomy of the Late Triassic phytosaur *Machaeroprosopus*, with the description of a new species from West Texas. *Earth Environ. Sci. Trans. R. Soc. Edinburgh* **103,** 269–312 (2013).

69. Kammerer, C. F., Butler, R. J., Bandyopadhyay, S. & Stocker, M. R. Relationships of the Indian phytosaur *Parasuchus hislopi* Lydekker, 1885. *Pap. Palaeontol.* **2,** 1–23 (2016).

70. Kimmig, J. & Arp, G. Phytosaur remains from the Norian Arnstadt Formation (Leine Valley, Germany), with reference to European phytosaur habitats. *Palaeodiversity* **3,** 215–224 (2010).

71. Butler, R. J. *et al.* New clade of enigmatic early archosaurs yields insights into early pseudosuchian phylogeny and the biogeography of the archosaur radiation. *BMC Evol. Biol.* **14,** 128 (2014).

72. von Baczko, M. B. & Ezcurra, M. D. Ornithosuchidae: a group of Triassic archosaurs with a unique ankle joint. *Geol. Soc. London, Spec. Publ.* **379,** 187–202 (2013).

73. Nesbitt, S. J. & Butler, R. J. Redescription of the archosaur *Parringtonia gracilis* from the Middle Triassic Manda beds of Tanzania, and the antiquity of Erpetosuchidae -- Corrigendum. *Geol. Mag.* **150,** 239 (2013).

74. Desojo, J. B. *et al.* Aetosauria: a clade of armoured pseudosuchians from the Upper Triassic continental beds. *Geol. Soc. London, Spec. Publ.* **379,** 203–239 (2013).

75. Small, B. J. & Martz, J. W. A new aetosaur from the Upper Triassic Chinle Formation of the Eagle Basin, Colorado, USA. *Geol. Soc. London, Spec. Publ.* **379,** 393–412 (2013).

76. Parker, W. G. Revised phylogenetic analysis of the Aetosauria (Archosauria: Pseudosuchia); assessing the effects of incongruent morphological character sets. *PeerJ* **4,** e1583 (2016).

77. Desojo, J. B., Ezcurra, M. D. & Kischlat, E. E. A new aetosaur genus (Archosauria: Pseudosuchia) from the early Late Triassic of southern Brazil. *Zootaxa* **3166**, 1–33 (2012).

78. Nesbitt, S. J. The early evolution of archosaurs: Relationships and the origin of major clades. *Bull. Am. Museum Nat. Hist.* **352,** 1–292 (2011).

79. Brusatte, S. L., Benton, M. J., Desojo, J. B. & Langer, M. C. The higher-level phylogeny of Archosauria (Tetrapoda: Diapsida). *J. Syst. Palaeontol.* **8,** 3–47 (2010).

80. Lautenschlager, S. & Desojo, J. B. Reassessment of the Middle Triassic rauisuchian archosaurs *Ticinosuchus ferox* and *Stagonosuchus nyassicus*. *Palaontologische Zeitschrift* **85,** 357–381 (2011).

81. Nesbitt, S. J., Barrett, P. M., Werning, S., Sidor, C. A. & Charig, A. J. The oldest dinosaur? A Middle Triassic dinosauriform from Tanzania. *Biol. Lett.* **9,** 20120949 (2013).

82. De Franca, M. A. G., Langer, M. C. & Ferigolo, J. The skull anatomy of *Decuriasuchus quartacolonia* (Pseudosuchia: Suchia: Loricata) from the middle

Triassic of Brazil. *Geol. Soc. London, Spec. Publ.* **379,** 469–501 (2013).

83. Lacerda, M. B., Schultz, C. L. & Bertoni-Machado, C. First `Rauisuchian' archosaur (Pseudosuchia, Loricata) for the Middle Triassic *Santacruzodon* Assemblage Zone (Santa Maria Supersequence), Rio Grande do Sul State, Brazil. *PLoS One* **10,** e0118563 (2015).

84. Irmis, R. B., Nesbitt, S. J. & Sues, H. D. Early Crocodylomorpha. *Geol. Soc. London, Spec. Publ.* **379,** 275–302 (2013).

85. Clark, J. M., Xing, X., Forster, C. A. & Yuan, W. A Middle Jurassic 'sphenosuchian' from China and the origin of the crocodylian skull. *Nature* **430,** 1021–2024 (2004).

86. Larsson, H. C. E. & Sues, H. D. Cranial osteology and phylogenetic relationships of Hamadasuchus rebouli (Crocodyliformes: Mesoeucrocodylia) from the Cretaceous of Morocco. *Zool. J. Linn. Soc.* **149,** 533–567 (2007).

87. Bronzati, M., Montefeltro, F. C. & Langer, M. C. A species-level supertree of Crocodyliformes. *Hist. Biol.* **24,** 598–606 (2012).

88. Zanno, L. E., Drymala, S., Nesbitt, S. J. & Schneider, V. P. Early crocodylomorph increases top tier predatory diversity during rise of dinosaurs. *Sci. Rep.* **5,** 1–6 (2015).

89. Barrett, P. M. & Xing, X. A reassessment of *Dianchungosaurus lufengensis* yang, 1982a, an enigmatic reptile from the Lower Lufeng Formation (lower Jurassic) of Yunnan Province, People's Republic of China. *J. Paleontol.* **79,** 981–986 (2005).

90. Ezcurra, M. D. A review of the systematic position of the dinosauriform archosaur *Eucoelophysis baldwini* Sullivan & Lucas, 1999 from the Upper Triassic of New Mexico, USA. *Geodiversitas* **28,** 649–684 (2006).

91. Benton, M. J. & Walker, A. D. Saltopus, a dinosauriform from the Upper Triassic of

Scotland. *Earth Environ. Sci. Trans. R. Soc. Edinburgh* **101,** 285–299 (2010).

92. Kammerer, C. F., Nesbitt, S. J. & Shubin, N. H. The first silesaurid dinosauriform from the Late Triassic of Morocco. *Acta Palaeontol. Pol.* **57,** 277–284 (2012).

93. Langer, M. C., Nesbitt, S. J., Bittencourt, J. S. & Irmis, R. B. Non-dinosaurian Dinosauromorpha. *Geol. Soc. London, Spec. Publ.* **379,** 157–186 (2013).

94. Small, B. J. A Late Triassic dinosauromorph assemblage from the Eagle Basin (Chinle Formation), Colorado, U.S.A. *J. Vertebr. Paleontol.* **29,** 182A (2009).

95. Andres, B. & Myers, T. S. Lone star pterosaurs. *Earth Environ. Sci. Trans. R. Soc. Edinburgh* **103,** 383–398 (2013).

96. Kellner, A. W. A. Comments on triassic pterosaurs with discussion about ontogeny and description of new taxa. *An. Acad. Bras. Cienc.* **87,** 669–689 (2015).

97. Dalla Vecchia, F. M. Triassic pterosaurs. *Geol. Soc. London, Spec. Publ.* **379,** 119–155 (2013).

98. Nesbitt, S. J. *et al.* Ecologically distinct dinosaurian sister group shows early diversification of Ornithodira. *Nature* **464,** 95–98 (2010).

99. Peecook, B. R. *et al.* A new silesaurid from the upper Ntawere Formation of Zambia (Middle Triassic) demonstrates the rapid diversification of Silesauridae (Avemetatarsalia, Dinosauriformes). *J. Vertebr. Paleontol.* **33,** 1127–1137 (2013).

100. Martínez, R. N. *et al.* Vertebrate succession in the Ischigualasto Formation. *J. Vertebr. Paleontol.* **32,** 10–30 (2012).

101. Bittencourt, J. D. S., Arcucci, A. B., Marsicano, C. A. & Langer, M. C. Osteology of the Middle Triassic archosaur *Lewisuchus admixtus* Romer (Chañares Formation,

Argentina), its inclusivity, and relationships amongst early dinosauromorphs. *J. Syst. Palaeontol.* **0,** 1–31 (2014).

102. Butler, R. J., Upchurch, P. & Norman, D. B. The phylogeny of the ornithischian dinosaurs. *J. Syst. Palaeontol.* **6,** 1–40 (2008).

103. Butler, R. J. *et al.* Lower limits of ornithischian dinosaur body size inferred from a new Upper Jurassic heterodontosaurid from North America. *Proc. Biol. Sci.* **277,** 375–381 (2010).

104. Barrett, P. M. *et al.* A palaeoequatorial ornithischian and new constraints on early dinosaur diversification. *Proc. R. Soc. Biol. Sci.* **281,** 20141147 (2014).

105. Porro, L. B., Butler, R. J., Barrett, P. M., Moore-Fay, S. & Abel, R. L. New heterodontosaurid specimens from the Lower Jurassic of southern Africa and the early ornithischian dinosaur radiation. *Earth Environ. Sci. Trans. R. Soc. Edinburgh* **101,** 351–366 (2010).

106. Pol, D., Rauhut, O. W. M. & Becerra, M. A Middle Jurassic heterodontosaurid dinosaur from Patagonia and the evolution of heterodontosaurids. *Naturwissenschaften* **98,** 369–379 (2011).

107. Butler, R. J. The 'fabrosaurid' ornithischian dinosaurs of the Upper Elliot Formation (Lower Jurassic) of South Africa and Lesotho. *Zool. J. Linn. Soc.* **145,** 175–218 (2005).

108. Irmis, R. B., Parker, W. G., Nesbitt, S. J. & Liu, J. Early ornithischian dinosaurs: the Triassic record. *Hist. Biol.* **19,** 3–22 (2007).

109. Norman, D. B., Butler, R. J. & Maidment, S. C. R. Reconsidering the status and affinities of the ornithischian dinosaur *Tatisaurus oehler*i Simmons, 1965. *Zool. J.*

Linn. Soc. **150,** 865–874 (2007).

110. Smith, N. D., Makovicky, P. J., Hammer, W. R. & Currie, P. J. Osteology of Cryolophosaurus ellioti (Dinosauria: Theropoda) from the Early Jurassic of Antarctica and implications for early theropod evolution. *Zool. J. Linn. Soc.* **151,** 377–421 (2007).

111. Ezcurra, M. D. & Brusatte, S. L. Taxonomic and phylogenetic reassessment of the early neotheropod dinosaur *Camposaurus arizonensis* from the Late Triassic of North America. *Palaeontology* **54,** 763–772 (2011).

112. Sues, H.-D., Nesbitt, S. J., Berman, D. S. & Henrici, A. C. A late-surviving basal theropod dinosaur from the latest Triassic of North America. *Proc. Biol. Sci.* **278,** 3459–64 (2011).

113. Hendrickx, C., Hartman, S. A. & Mateus, O. An Overview of Non-Avian Theropod Discoveries and Classification. *PalArch's J. Vertebr. Palaeontol.* **12,** 1–73 (2015).

114. You, H. L., Azuma, Y., Wang, T., Wang, Y. M. & Dong, Z. M. The first well-preserved coelophysoid theropod dinosaur from Asia. *Zootaxa* **3873,** 233–249 (2014).

115. Nesbitt, S. J., Irmis, R. B. & Parker, W. G. A critical re-evaluation of the Late Triassic dinosaur taxa of North America. *J. Syst. Palaeontol.* **5,** 209–243 (2007).

116. Carrano, M. T., Hutchinson, J. R. & Sampson, S. D. New information on *Segisaurus halli*, a small theropod dinosaur from the Early Jurassic of Arizona. *J. Vertebr. Paleontol.* **25,** 835–849 (2005).

117. Ezcurra, M. & Cuny, G. The coelophysoid *Lophostropheus airelensis*, gen. nov.: a review of the systematics of '*Liliensternus*' *airelensis* from the Triassic–Jurassic outcrops of Normandy (France). *J. Vertebr. Paleontol.* **27,** 73–86 (2007).

118. Yates, A. M. A new theropod dinosaur from the Early Jurassic of South Africa and its

implications for the early evolution of theropods. *Palaeontol. Africana* **41,** 105–122 (2005).

119. Kutty, T. S., Chatterjee, S., Galton, P. M. & Upchurch, P. Basal sauropodomorphs (Dinosauria : Saurischia) from the lower Jurassic of India: Their anatomy and relationships. *J. Paleontol.* **81,** 1218–1240 (2007).

120. Niedźwiedzki, G., Brusatte, S. L., Sulej, T. & Butler, R. J. Basal dinosauriform and theropod dinosaurs from the mid-late Norian (Late Triassic) of Poland: Implications for Triassic dinosaur evolution and distribution. *Palaeontology* **57,** 1121–1142 (2014).

121. Barrett, P. M. The affinities of the enigmatic dinosaur *Eshanosaurus deguchiianus* from the Early Jurassic of Yunnan Province, People's Republic of China. *Palaeontology* **52,** 681–688 (2009).

122. Allain, R. & Aquesbi, N. Anatomy and phylogenetic relationships of *Tazoudasaurus naimi* (Dinosauria, Sauropoda) from the late Early Jurassic of Morocco. *Geodiversitas* **30,** 345–424 (2008).

123. Apaldetti, C., Martinez, R. N., Alcober, O. A. & Pol, D. A new basal sauropodomorph (Dinosauria: Saurischia) from Quebrada del Barro formation (Marayes-El carrizal basin), Northwestern Argentina. *PLoS One* **6,** e26964 (2011).

124. Apaldetti, C., Pol, D. & Yates, A. The postcranial anatomy of *Coloradisaurus brevis* (Dinosauria: Sauropodomorpha) from the Late Triassic of Argentina and its phylogenetic implications. *Palaeontology* **56,** 277–301 (2013).

125. Apaldetti, C., Martinez, R. N., Pol, D. & Souter, T. Redescription of the skull of *Coloradisaurus brevis* (Dinosauria, Sauropodomorpha) from the Late Triassic Los Colorados Formation of the Ischigualasto-Villa Union Basin, northwestern Argentina.

J. Vertebr. Paleontol. **34,** 1113–1132 (2014).

126. Benson, R. B. J. *et al.* Rates of Dinosaur Body Mass Evolution Indicate 170 Million Years of Sustained Ecological Innovation on the Avian Stem Lineage. *PLoS Biol.* **12**, e1001896 (2014).

127. Martinez, R. N. *et al.* A basal dinosaur from the dawn of the dinosaur era in southwestern Pangaea. *Science* **331,** 206–210 (2011).

128. Martínez, R. N., Apaldetti, C. & Abelin, D. Basal sauropodomorphs from the Ischigualasto Formation. *J. Vertebr. Paleontol.* **32,** 51–69 (2012).

129. Mcphee, B. W., Yates, A. M., Choiniere, J. N. & Abdala, F. The complete anatomy and phylogenetic relationships of *Antetonitrus ingenipes* (Sauropodiformes, Dinosauria): Implications for the origins of Sauropoda. *Zool. J. Linn. Soc.* **171,** 151–205 (2014).

130. McPhee, B. W., Bonnan, M. F., Yates, A. M., Neveling, J. & Choiniere, J. N. A new basal sauropod from the pre-Toarcian Jurassic of South Africa: evidence of niche-partitioning at the sauropodomorph–sauropod boundary? *Sci. Rep.* **5**, 13224 (2015).

131. McPhee, B. W., Choiniere, J. N., Yates, A. M. & Viglietti, P. A. A second species of *Eucnemesaurus* Van Hoepen, 1920 (Dinosauria, Sauropodomorpha): new information on the diversity and evolution of the sauropodomorph fauna of South Africa's lower Elliot Formation (latest Triassic). *J. Vertebr. Paleontol.* **35,** e980504 (2015).

132. Novas, F. E., Ezcurra, M. D., Chatterjee, S. & Kutty, T. S. New dinosaur species from the Upper Triassic Upper Maleri and Lower Dharmaram formations of Central India. *Earth Environ. Sci. Trans. R. Soc. Edinburgh* **101,** 333–349 (2010).

133. Otero, A. & Pol, D. Postcranial anatomy and phylogenetic relationships of *Mussaurus*

patagonicus (Dinosauria, Sauropodomorpha). *J. Vertebr. Paleontol.* **33,** 1138–1168 (2013).

134. Otero, A., Krupandan, E., Pol, D., Chinsamy, A. & Choiniere, J. A new basal sauropodiform from South Africa and the phylogenetic relationships of basal sauropodomorphs. *Zool. J. Linn. Soc.* **174,** 589–634 (2015).

135. Yates, A. M. & Kitching, J. W. The earliest known sauropod dinosaur and the first steps towards sauropod locomotion. *Proc. Biol. Sci.* **270,** 1753–8 (2003).

136. Yates, A. M., Bonnan, M. F., Neveling, J., Chinsamy, A. & Blackbeard, M. G. A new transitional sauropodomorph dinosaur from the Early Jurassic of South Africa and the evolution of sauropod feeding and quadrupedalism. *Proc. Biol. Sci.* **277,** 787–794 (2010).

137. Galton, P. M. & Kermack, D. The anatomy of *Pantydraco caducus,* a very basal sauropodomorph dinosaur from the Rhaetian (Upper Triassic) of South Wales UK. *Rev. Paleobiol.* **29,** 341–404 (2010).

138. Yates, A. M. A definite prosauropod dinosaur from the Lower Elliot Formation (Norian: Upper Triassic) of South Africa. *Palaeontol. Africana* 63–68 (2003).

139. Yates, A. M. Solving a dinosaurian puzzle: the identity of *Aliwalia rex* Galton. *Hist. Biol.* **19,** 93–123 (2007).

140. van Heerden, J. The morphology and taxonomy of *Euskelosaurus* (Reptilia: Saurischia; Late Triassic) from South Africa. *Navorsinge van die Nas. Museum, Bloemfontein* **4,** 21–84 (1979).

141. Galton, P. M. & Upchurch, P. in *The Dinosauria* (eds. Weishampel, D. B., Dodson, P. & Osmolska, H.) 232–258 (University of California Press, 2004).

142. Upchurch, P., Galton, P. M. & Barrett, P. M. A phylogenetic analysis of basal sauropodomorph relationships: implications for the origin of sauropod dinosaurs. *Spec. Pap. Palaeontol.* **77,** 57–90 (2007).

143. Barrett, P. M. A New Basal Sauropodomorph Dinosaur from the Upper Elliot Formation (Lower Jurassic) of South Africa. *J. Vertebr. Paleontol.* **29,** 1032–1045 (2009).

144. Prieto-Márquez, A. & Norell, M. A. Redescription of a nearly complete skull of *Plateosaurus* (Dinosauria: Sauropodomorpha) from the Trossingen (Germany). *Am. Museum Novit.* **3727,** 1–58 (2011).

145. Yates, A. M. The species taxonomy of the sauropodomorph dinosaurs from the Löwenstein formation (Norian , Late Triassic) of Germany. *Palaeontology* **46,** 317–337 (2003).

146. Galton, P. M. Bones of large dinosaurs (Prosauropoda and Stegosauria) from the Rhaetic Bone Bed (Upper Triassic) of Aust Cliff, southwest England. *Rev. Paleobiol.* **24,** 51–74 (2005).

147. Sekiya, T., Jin, X., Zheng, W., Shibata, M. & Azuma, Y. A new juvenile specimen of *Yunnanosaurus robustus* (Dinosauria: Sauropodomorpha) from Early to Middle Jurassic of Chuxiong Autonomous Prefecture, Yunnan Province, China. *Hist. Biol.* **26**, 252-277 (2014).

148. Sekiya, T. A new prosauropod dinosaur from Lower Jurassic in Lufeng of Yunnan. *Global Geology* **29**, 6-15 (2010).

149. Upchurch, P., Barrett, P. M., Xijin, Z. & Xing, X. A re-evaluation of *Chinshakiangosaurus chunghoensis* Ye vide Dong 1992 (Dinosauria,

Sauropodomorpha): Implications for cranial evolution in basal sauropod dinosaurs. *Geol. Mag.* **144,** 247 (2007).

150. Benson, R. B. J. Interrelationships of basal synapsids: cranial and postcranial morphological partitions suggest different topologies. *J. Syst. Palaeontol.* **2019,** 1–24 (2012).

151. Liu, J., Rubidge, B. S. & Li, J. New basal synapsid supports Laurasian origin for therapsids. *Acta Palaeontol. Pol.* **54,** 393–400 (2009).

152. Sidor, C. A. & Smith, R. M. H. A second burnetiamorph therapsid from the Permian Teekloof Formation of South Africa and its associated fauna. *J. Vertebr. Paleontol.* **27,** 420–430 (2007).

153. Kruger, A., Rubidge, B. S., Abdala, F. A., Chindebvu, E. G. & Jacobs, L. L. *Lende chiweta*, a new therapsid from Malawi, and its influence on burnetiamorph phylogeny and biogeography. *J. Vertebr. Paleontol.* **35,** e1008698 (2015).

154. Battail, B. & Surkov, Mikhail, V. in *The age of dinosaurs in Russia and Mongolia* (eds. Benton, M. J., Shishkin, M. A., Unwin, D. M. & Kurochkin, E. N.) 120–139 (Cambridge University Press, 2000).

155. Ivakhnenko, M. F. Biarmosuches from the Ocher Faunal Assemblage of Eastern Europe. *Paleontol. J.* **33,** 289–296 (1999).

156. Kammerer, C. F. Systematics of the Anteosauria (Therapsida: Dinocephalia). *J. Syst. Palaeontol.* **9,** 261–304 (2011).

157. Boonstra, L. D. The fauna of the *Tapinocephalus* Zone (Beaufort beds of the Karoo). *Ann. South African Museum* **56**, 1-73 (1969)..

158. Kammerer, C. F. & Sidor, C. A. Replacement names for the therapsid genera

Criocephalus Broom 1928 and *Olivieria* Brink 1965. *Palaeontol. africana* **38,** 71–72 (2002).

159. Liu, J. Osteology, ontogeny, and phylogenetic position of *Sinophoneus yumenensis* (Therapsida, Dinocephalia) from the Middle Permian Dashankou Fauna of China. *J. Vertebr. Paleontol.* **33,** 1394–1407 (2013).

160. Kammerer, C. F., Fröbisch, J. & Angielczyk, K. D. On the validity and phylogenetic position of *Eubrachiosaurus browni*, a kannemeyeriiform dicynodont (Anomodontia) from Triassic North America. *PLoS One* **8,** e64203 (2013).

161. Angielczyk, K. D., Rubidge, B. S., Day, M. O. & Lin, F. A reevaluation of *Brachyprosopus broomi* and *Chelydontops altidentalis* , dicynodonts (Therapsida, Anomodontia) from the middle Permian *Tapinocephalus* Assemblage Zone of the Karoo Basin, South Africa. *J. Vertebr. Paleontol.* **4634,** e1078342 (2016).

162. Kammerer, C. F., Angielczyk, K. D. & Fröbisch, J. Redescription of *Digalodon rubidgei*, an emydopoid dicynodont (Therapsida, Anomodontia) from the Late Permian of South Africa. *Foss. Rec.* **18,** 43–55 (2015).

163. Wyllie, A. A review of Robert Broom's therapsid holotypes: Have they survived the test of time? *Palaeontol. Africana* **39**, 1–19 (2003).

164. Fröbisch, J. Composition and similarity of global anomodont-bearing tetrapod faunas. *Earth-Science Rev.* **95,** 119–157 (2009).

165. Cox, C. B. On the palate, dentition, and classification of the fossil reptile *Endothiodon* and related genera. *Am. Museum Novit.* **2171,** 1–25 (1964).

166. Boos, A. D. S., Schultz, C. L., Vega, C. S. & Aumond, J. J. On the presence of the late Permian dicynodont *Endothiodon* in Brazil. *Palaeontology* **56,** 837–848 (2013).

167. Cox, C. B. & Angielczyk, K. D. A new endothiodont dicynodont (Therapsida, Anomodontia) from the Permian Ruhuhu Formation (Songea Group) of Tanzania and its feeding system. *J. Vertebr. Paleontol.* **35,** e935388 (2015).

168. Angielczyk, K. D. & Sullivan, C. *Diictodon feliceps* (Owen, 1876), a dicynodont (Therapsida, Anomodontia) species with a Pangaean distribution. *J. Vertebr. Paleontol.* **28,** 788–802 (2008).

169. Angielczyk, K. D. *et al.* New dicynodonts (Therapsida, Anomodontia) and updated tetrapod stratigraphy of the Permian Ruhuhu Formation (Songea Group, Ruhuhu Basin) of southern Tanzania. *J. Vertebr. Paleontol.* **34,** 1408–1426 (2014).

170. Angielczyk, K. D., Sidor, C. A., Nesbitt, S. J., Smith, R. M. H. & Tsuji, L. A. Taxonomic revision and new observations on the postcranial skeleton, biogeography, and biostratigraphy of the dicynodont genus *Dicynodontoides*, the senior subjective synonym of *Kingoria* (Therapsida, Anomodontia). *J. Vertebr. Paleontol.* **29,** 1174–1187 (2009).

171. Kammerer, C. F., Angielczyk, K. D. & Fröbisch, J. A comprehensive taxonomic revision of *Dicynodon* (Therapsida, Anomodontia) and its implications for dicynodont phylogeny, biogeography, and biostratigraphy. *J. Vertebr. Paleontol.* **31,** 1–158 (2011).

172. Govender, R. Description of the postcranial anatomy of *Aulacephalodon baini* and its possible relationship with ‘*Aulacephalodon peavoti*’. *S. Afr. J. Sci.* **104,** 479–486 (2008).

173. Grine, F. E., Forster, C. A., Cluver, M. A. & Georgi, J. A. in *Amniote paleobiology - perspectives on the evolution of mammals, birds and reptiles.* (eds. Carrano, M. T., Gaudin, T. J., Blob, R. W. & Wible, J. R.) 432–503 (University of Chicago Press,

2006).

174. Damiani, R., Vasconcelos, C., Renaut, A., Hancox, J. & Yates, A. *Dolichuranus primaevus* (Therapsida: Anomodontia) from the Middle Triassic of Namibia and its phylogenetic relationships. *Palaeontology* **50,** 1531–1546 (2007).

175. Keyser, A. W. & Cruickshank, A. R. I. The origins and classification of Triassic dicynodonts. *Trans. Geol. Soc. South Africa* **82,** 81–108 (1979).

176. Lucas, S. G. & Hunt, A. P. A dicynodont from the Upper Triassic of New Mexico and its biochronological significance. *New Mex. Museum Nat. Hist. Sci. Bull.* **3,** 321–325 (1993).

177. Maisch, M. W. & Matzke, A. T. *Sungeodon kimkraemerae* n. gen. n. sp., the oldest kannemeyeriiform (Therapsida, Dicynodontia) and its implications for the early diversification of large herbivores after the P/T boundary. *Neues Jahrb. für Geol. und Paläontologie - Abhandlungen* **272,** 1–12 (2014).

178. Broili, F. Ein Fund von cf. *Placerias* Lucas in der kontinnentalen Trias von Europa. *Zentralblatt für Mineral. Geol. und Paläontologie* **1921,** 339–343 (1921).

179. Camp, C. L. & Welles, S. P. Triassic dicynodont reptiles. Part I. The North American genus *Placerias*. *Mem. Univ. Calif.* **13,** 255–304 (1956).

180. Lucas, S. G. & Wild, R. A Middle Triassic dicynodont from Germany and the biochronology of Triassic dicynodonts. *Stuttgarter Beiträge für Naturkunde, Ser. B* **220,** 1–16 (1995).

181. E. V. I. Gebauer, Phylogeny and evolution of the Gorgonopsia with a special reference to the skull and skeleton of GPIT/RE/7113 (‘*Aelurognathus*?’ *parringtoni*), Universität Tübingen (2007).

182. Kammerer, C. F. Systematics of the Rubidgeinae (Therapsida: Gorgonopsia). *1. PeerJ* **4,** e1608 (2016).

183. Kammerer, C. F. Cranial osteology of *Arctognathus curvimola*, a short-snouted gorgonopsian from the Late Permian of South Africa. *Pap. Palaeontol.* **1,** 41–58 (2015).

184. Ivakhnenko, M. F. Cranial morphology and evolution of Permian Dinomorpha (Eotherapsida) of eastern Europe. *Paleontol. J.* **42,** 859–995 (2008).

185. Efremov, I. A. [The terrestrial vertebrate fauna from the Permian copper sandstones of the western Fore-Urals]. *Tr. Paleontol. Instituta, Akad. Nauk SSSR* **54,** 1–416 (1954). [In Russian]

186. Huttenlocker, A. K. Body size reductions in nonmammalian eutheriodont therapsids (Synapsida) during the end-permian mass extinction. *PLoS One* **9,** e87553 (2014).

187. Huttenlocker, A. K., Sidor, C. A. & Angielczyk, K. D. A new eutherocephalian (Therapsida, Therocephalia) from the upper Permian Madumabisa Mudstone Formation (Luangwa Basin) of Zambia. *J. Vertebr. Paleontol.* **35,** e969400 (2015).

188. Abdala, F., Kammerer, C. F., Day, M. O., Jirah, S. & Rubidge, B. S. Adult morphology of the therocephalian *Simorhinella baini* from the middle Permian of South Africa and the taxonomy, paleobiogeography, and temporal distribution of the Lycosuchidae. *J. Paleontol.* **88,** 1139–1153 (2014).

189. Huttenlocker, A. An investigation into the cladistic relationships and monophyly of therocephalian therapsids (Amniota: Synapsida). *Zool. J. Linn. Soc.* **157,** 865–891 (2009).

190. Huttenlocker, A. K., Sidor, C. a. & Smith, R. M. H. A new specimen of

Promoschorhynchus (Therapsida: Therocephalia: Akidnognathidae) from the Lower Triassic of South Africa and its implications for theriodont survivorship across the Permo-Triassic boundary. *J. Vertebr. Paleontol.* **31,** 405–421 (2011).

191. A. K. Huttenlocker, C. A. Sidor, Taxonomic revision of therocephalians (Therapsida: Theriodontia) from the Lower Triassic of Antarctica **3738**, 1–20 (2012).

192. Sidor, C. A. *et al.* Provincialization of terrestrial faunas following the end-Permian mass extinction. *Proc. Natl. Acad. Sci. U. S. A.* **110,** 8129–33 (2013).

193. Huttenlocker, A. K. & Botha-Brink, J. Bone microstructure and the evolution of growth patterns in Permo-Triassic therocephalians (Amniota, Therapsida) of South Africa. *PeerJ* **2,** 1–31 (2014).

194. Ivakhnenko, M. F. The first Whaitsiid (Therocephalia, Theromorpha) from the terminal Permian of eastern Europe. *Paleontol. J.* **42,** 409–413 (2008).

195. Ruta, M., Botha-Brink, J., Mitchell, S.A & Benton, M. J. The radiation of cynodonts and the ground plan of mammalian morphological diversity. *Proc. Biol. Sci.* **280,** 20131865 (2013).

196. Abdala, F. & Ribeiro, A. M. A new therioherpetid cynodont from the Santa Maria Formation (middle Late Triassic), southern Brazil. *Geodiversitas* **22,** 589–596 (2000).

197. Liu, J. The taxonomy of the traversodontid cynodonts *Exaeretodon* and *Ischignathus*. *Rev. Bras. Paleontol.* **10,** 133–136 (2007).

198. Liu, J. & Sues, H.-D. Dentition and tooth replacement of *Boreogomphodon* (Cynodontia: Traversodontidae) from the Upper Triassic of North Carolina, USA. *Vertebr. Palasiat.* **48,** 169–184 (2010).

199. Luo, Z.-X., Gatesy, S. M., Jenkins, F. A., Amaral, W. W. & Shubin, N. H. Mandibular

and dental characteristics of Late Triassic mammaliaform *Haramiyavia* and their ramifications for basal mammal evolution. *Proc. Natl. Acad. Sci.* **112,** E7101–E7109 (2015).

200. Ray, S. A new Late Triassic traversodontid cynodont (Therapsida, Eucynodontia) from India. *J. Vertebr. Paleontol.* **35,** e930472 (2015).

201. Liu, J. & Olsen, P. The phylogenetic relationships of Eucynodontia (Amniota: Synapsida). *J. Mamm. Evol.* **17,** 151–176 (2010).

202. Soares, M. B., Martinelli, A. G. & De Oliveira, T. V. A new prozostrodontian cynodont (Therapsida) from the Late Triassic *Riograndia* Assemblage Zone (Santa Maria Supersequence) of southern Brazil. *An. Acad. Bras. Cienc.* **86,** 1673–1691 (2014).

203. de Oliveira, T. V., Martinelli, A. G. & Soares, M. B. New information about *Irajatherium hernandezi* Martinelli, Bonaparte, Schultz & Rubert 2005 (Eucynodontia, Tritheledontidae) from the upper triassic (Caturrita Formation, Paraná Basin) of Brazil. *Palaontologische Zeitschrift* **85,** 67–82 (2011).

204. Godefroit, P. New traversodontid (Therapsida: Cynodontia) teeth from the Upper Triassic of Habay-la-Vieille (southern Belgium). *Paläontologische Zeitschrift* **73,** 385–394 (1999).

205. Sues, H.-D. On *Microconodon*, a Late Triassic cynodont from the Newark Supergroup of Eastern North America. *Bull. Museum Comp. Zool.* **156,** 37–48 (2001).

206. Kammerer, C. F. & Abdala, F. *Procynosuchus* Broom, 1937 (Therapsida, Cynodontia): proposed precedence over *Cyrbasiodon* Broom, 1931 and *Parathrinaxodon* Parrington, 1936. *Bull. Zool. Nomencl.* **66,** 64–69 (2009).

207. Abdala, F., Hancox, P. J. & Neveling, J. Cynodonts from the Uppermost Burgersdorp Formation, South Africa, and their bearing on the biostratigraphy and correlation of the Triassic *Cynognathus* Assemblage Zone. *J. Vertebr. Paleontol.* **25,** 192–199 (2005).

208. Sidor, C. A. & Smith, R. M. H. A new galesaurid (Therapsida: Cynodontia) from the Lower Triassic of South Africa. *Palaeontology* **47,** 535–556 (2004).

209. Bonaparte, J. J. F. Evolution of the Brasilodontidae (Cynodontia-Eucynodontia). *Hist. Biol.* **2963,** 1–11 (2012).

210. Martinelli, A. G., de la Fuente, M. S. & Abdala, F. *Diademodon tetragonus* Seeley, 1894 (Therapsida: Cynodontia) in the Triassic of South America and its biostratigraphic implications . *J. Vertebr. Paleontol.* **29,** 852–862 (2009).

211. Abdala, F. & Giannini, N. P. Chiniquodontid cynodonts: Systematic and morphometric considerations. *Palaeontology* **45,** 1151–1170 (2002).

212. Lucas, S. G. & Luo, Z.-X. *Adelobasileus* from the Upper Triassic of West Texas : The oldest mammal. *J. Vertebr. Paleontol.* **13,** 309–334 (1993).

213. Abdala, F. & Malabarba, M. C. Enamel microstructure in *Exaeretodon*, a Late Triassic South American traversodontid (Therapsida: Cynodontia). *Rev. Bras. Paleontol.* **10,** 71–78 (2007).

214. Datta, P. M., Das, D. P. & Luo, Z. X. A Late Triassic dromatheriid (Synapsida : Cynodontia) from India. *Ann. Carnegie Museum* **73,** 72–84 (2004).

215. Oliveira, É. V. Reevaluation of *Therioherpeton cargnini* Bonaparte & Barberena, 1975 (Probainognathia, Therioherpetidae) from the upper triassic of Brazil. *Geodiversitas* **28,** 447–465 (2006).

216. Fedak, T. J., Sues, H.-D., Olsen, P. E. & Gardner, J. First record of the tritylodontid

cynodont *Oligokyphus* and cynodont postcranial bones from the McCoy Brook Formation of Nova Scotia, Canada. *Can. J. Earth Sci.* **52,** 244–249 (2015).

217. Montellano-Ballesteros, M., Hopson, J. A. & Clark, J. M. Late Early Jurassic mammaliaforms from Huizachal Canyon, Tamaulipas, México. *J. Vertebr. Paleontol.* **28,** 1130–1143 (2008).

218. Gao, C.-L. *et al.* A new mammal skull from the Lower Cretaceous of China with implications for the evolution of obtuse-angled molars and 'amphilestid' eutriconodonts. *Proc. R. Soc. B Biol. Sci.* **277,** 237–246 (2010).

219. Gaetano, L. C. & Rougier, G. W. New materials of *Argentoconodon fariasorum* (Mammaliaformes, Triconodontidae) from the Jurassic of Argentina and its bearing on triconodont phylogeny. *J. Vertebr. Paleontol.* **31,** 829–843 (2011).

220. Gaetano, L. C. & Rougier, G. W. First Amphilestid from South America: A Molariform from the Jurassic Cañadón Asfalto Formation, Patagonia, Argentina. *J. Mamm. Evol.* **19,** 235–248 (2012).

221. Rougier, G. W., Martinelli, A. G., Forasiepi, A. M. & Novacek, M. J. New Jurassic mammals from Patagonia, Argentina: A reappraisal of australosphenidan morphology and interrelationships. *Am. Museum Novit.* **3566,** 1 (2007).

222. Rougier, G. W., Apesteguía, S. & Gaetano, L. C. Highly specialized mammalian skulls from the Late Cretaceous of South America. *Nature* **479,** 98–102 (2011).

223. Luo, Z.-X. A New Mammaliaform from the Early Jurassic and Evolution of Mammalian Characteristics. *Science* **292,** 1535–1540 (2001).

224. Prasad, G. V. R. & Manhas, B. K. First docodont mammals of Laurasian affinity from India. *Curr. Sci.* **81,** 1235–1238 (2001).

225. Sigogneau-Russell, D. Two possibly aquatic triconodont mammals from the Early Cretaceous of Morocco. *Acta Palaeontol. Pol.* **40,** 149–162 (1995).

226. Hahn, G., Sigogneau-Russell, D. & Godefroit, P. New data on *Brachyzostrodon* (Mammalia; Upper Triassic). *Geol. Palaeontol.* **25,** 237–249 (1991).

227. Clemens, W. A. A problem in morganucodontid taxonomy (Mammalia). *Zool. J. Linn. Soc.* **66,** 1–14 (1979).

228. Clemens, W. A. New morganucodontans from an Early Jurassic fissure filling in Wales (United Kingdom). *Palaeontology* **54,** 1139–1156 (2011).

229. Butler, P. M. Review of the early allotherian mammals. *Acta Palaeontol. Pol.* **45,** 317–342 (2000).

230. Parmar, V., Prasad, G. V. R. & Kumar, D. The first multituberculate mammal from India. *Naturwissenschaften* **100,** 515–523 (2013).

231. M. Carrano, R. J. Butler, R. Benson, T. Liebrecht *et al*. (2016) Taxonomic occurrences of Lopingian to Toarcian recorded in the *Paleobiology Database*. https://paleobiodb.org.

232. Muttoni, G. *et al.* Tethyan magnetostratigraphy from Pizzi Mondello (Sicily) and correlation to the Late Triassic Newark astrochronological polarity time scale Tethyan magnetostratigraphy from Pizzo Mondello (Sicily) and correlation to the Late Triassic Newark astrochron. *Geol. Soc. Am. Bull.* **116,** 1043–1058 (2004).

233. Irmis, R. B., Martz, J. W., Parker, W. G. & Nesbitt, S. J. Re-evaluating the correlation between Late Triassic terrestrial vertebrate biostratigraphy and the GSSP-defined marine stages. *Albertiana* **38,** 40–52 (2009).

234. Olsen, P. E., Kent, D. V & Whiteside, J. H. Implications of the Newark Supergroup-

based astrochronology and geomagnetic polarity time scale (Newark-APTS) for the tempo and mode of the early diversification of the Dinosauria. **2011,** 201–229 (2011).

235. Kent, D. V., Malnis, P. S., Colombi, C. E., Alcober, O. A. & Martinez, R. N. Age constraints on the dispersal of dinosaurs in the Late Triassic from magnetochronology of the Los Colorados Formation (Argentina). *Proc. Natl. Acad. Sci.* **111,** 7958–7963 (2014).

236. Lucas, S. G. The Triassic timescale based on nonmarine tetrapod biostratigraphy and biochronology. *Geol. Soc. London, Spec. Publ.* **334,** 447–500 (2010).

237. Ramezani, J. *et al.* High-precision U-Pb zircon geochronology of the late triassic chinle formation, Petrififed Forest National Park (Arizona, USA): Temporal constraints on the early evolution of dinosaurs. *Bull. Geol. Soc. Am.* **123,** 2142–2159 (2011).

238. Lucas, S. G. Global Triassic tetrapod biostratigraphy and biochronology. *Palaeogeogr. Palaeoclimatol. Palaeoecol.* **143,** 347–384 (1998).

239. Tourani, A., Lund, J. J., Benaouiss, N. & Gaupp, R. Stratigraphy of Triassic syn-rift deposition in Western Morocco. *Zentralblatt für Mineral. Geol. und Paläontologie* **1998,** 1193–1215 (2000).

240. Sues, H.-D. & Fraser, N. C. *Triassic life on land: The great transition*. (Columbia University Press, 2010).

241. Bachmann, G. H. & Kozur, H. W. The Germanic Triassic: Correlations with the international chronostratigraphic scale , numerical ages and Milankovitch cyclicity. *Hallersches Jahrb. Geowiss.* **26,** 17–62 (2004).

242. Bachmann, G. H. & Kozur, H. W. Updated correlation of the Germanic Triassic with

the Tethyan scale. *Berichte der Geol. Bundesanstalt* **76,** 53–58 (2008).

243. Muttoni, G. *et al.* Rhaetian magneto-biostratigraphy from the Southern Alps (Italy): Constraints on Triassic chronology. *Palaeogeogr. Palaeoclimatol. Palaeoecol.* **285,** 1–16 (2010).

244. Lucas, S. G., Tanner, L. H., Kozur, H. W., Weems, R. E. & Heckert, A. B. The Late Triassic timescale: Age and correlation of the Carnian-Norian boundary. *Earth-Science Rev.* **114,** 1–18 (2012).

245. Szulc, J. Sedimentary environments of the vertebrate-bearing Norian deposits from Krasiejow, Upper Silesia (Poland). *Hallesches Jahrb. Geowiss.* **19,** 161–170 (2005).

246. Dzik, J. & Sulej, T. A review of the early Late Triassic Krasiejow biota from Silesia, Poland. *Palaeontol. Pol.* **64,** 3–27 (2007).

247. Lucas, S. G., Spielmann, J. A. & Hunt, A. P. Biochronological significance of Late Triassic tetrapods from Krasiejow, Poland. *Glob. Triassic, New Mex. Museum Nat. Hist. Sci. Bull.* **41,** 241–258 (2007).

248. Horn, B. L. D. *et al.* A new third-order sequence stratigraphic framework applied to the Triassic of the Paraná Basin, Rio Grande do Sul, Brazil, based on structural, stratigraphic and paleontological data. *J. South Am. Earth Sci.* **55,** 123–132 (2014).

249. Langer, M. C., Ribeiro, A. M., Schultz, C. L. & Ferigolo, J. The continental tetrapod-bearing Triassic of South Brazil. *Glob. Triassic* **41,** 201–218 (2007).

250. Bonaparte, J. F. El Mesozoico de America del Sur y sus tetrapodos. *Opera Lilloana* **26,** 1–596 (1978).

251. Wang, Z. S., Rasbury, E. T., Hanson, G. N. & Meyers, W. J. Using the U-Pb system of calcretes to date the time of sedimentation of clastic sedimentary rocks. *Geochim.*

Cosmochim. Acta **62,** 2823–2835 (1998).

252. Hüsing, S. K., Deenen, M. H. L., Koopmans, J. G. & Krijgsman, W. Magnetostratigraphic dating of the proposed Rhaetian GSSP at Steinbergkogel (Upper Triassic, Austria): Implications for the Late Triassic time scale. *Earth Planet. Sci. Lett.* **302,** 203–216 (2011).

253. Roghi, G., Mietto, P. & Dalla Vecchia, F. M. Contribution of the conodont biostratigraphy of the dolomia di forni (Upper triassic, Carnia, NE Italy). *Mem. di Sci. Geol.* **47,** 125–133 (1995).

254. Donofrio, D. A., Brandner, R. & Poleschinski, W. Conodonten der Seefeld-Formation: Ein Beitrag zur Bio- und Lithostratigraphie der Hauptdolomit-Plattform (Obertrias, Westliche Nördliche Kalkalpen, Tirol). *Geol. Mitteilungen Innsbruck* **26,** 91–107 (2003).

255. Moix, P., Kozur, H. W., Stampfli, G. M. & Mostler, H. New palaeontological, biostratigraphical and palaeogeographical results from the Triassic of the Mersin mélange, SE Turkey. *Glob. Triassic, New Mex. Museum Nat. Hist. Sci. Bull.* **41,** 282–311 (2007).

256. Martínez, R. N., Apaldetti, C., Correa, G. A. & Abelín, D. A Norian lagerpetid dinosauromorph from the Quebrada del Barro Formation, northwestern Argentina. *Ameghiniana* **53,** 1–13 (2016).

257. Knoll, F. Review of the tetrapod fauna of the 'Lower Stormberg Group' of the main Karoo Basin (southern Africa): Implication for the age of the Lower Elliot Formation. *Bull. la Soc. Geol. Fr.* **175,** 73–83 (2004).

258. Shubin, N. H. & Sues, H.-D. Biogeography of early Mesozoic continental tetrapods:

patterns and implications. *Paleobiology* **17,** 214–230 (1991).

259. Upchurch, P., Hunn, C. A. & Norman, D. B. An analysis of dinosaurian biogeography: evidence for the existence of vicariance and dispersal patterns caused by geological events. *Proc. R. Soc. B Biol. Sci.* **269,** 613–21 (2002).

260. Ezcurra, M. D. Biogeography of Triassic tetrapods: Evidence for provincialism and driven sympatric cladogenesis in the early evolution of modern tetrapod lineages. *Proc. Biol. Sci.* **277,** 2547–2552 (2010).

261. Dunhill, A. M., Bestwick, J., Narey, H. & Sciberras, J. Dinosaur biogeographical structure and Mesozoic continental fragmentation: A network-based approach. *J. Biogeogr.* **43,** 1691–1704 (2016).

262. Fraser, N. C. & Sues, H.-D. The beginning of the 'Age of Dinosaurs': A brief overview of terrestrial biotic changes during the Triassic. *Earth Environ. Sci. Trans. R. Soc. Edinburgh* **101,** 189–200 (2010).

263. Soul, L. C. & Lloyd, G. T. dispeRse: Models For Paleobiogeography. https://github.com/laurasoul/dispeRse

264. Y. Sun *et al.* Lethally high temperatures during the Early Triassic greenhuse. *Science* **338**, 366-370 (2012).

265. Sidor, C. A. *et al.* Permian tetrapods from the Sahara show climate-controlled endemism in Pangaea. *Nature* **434,** 886–889 (2005).

266. Bambach, R. K. Phanerozoic Biodiversity Mass Extinctions. *Annu. Rev. Earth Planet. Sci.* **34,** 127–155 (2006).
