# Supplementary material for: Mass extinctions drove increased global faunal cosmopolitanism on the supercontinent Pangaea
Source: Nat Commun. 2017 Oct 10;8:733. doi: 10.1038/s41467-017-00827-7 (PMC5635108; doi:10.1038/s41467-017-00827-7)
Supplement: Supplementary file 3 — Description of Additional Supplementary Files [file 41467_2017_827_MOESM3_ESM.pdf]

Description of Additional Supplementary Files

File Name: Supplementary Data 1
Description: Nexus file of the amniote informal supertree used in this study.

File Name: Supplementary Data 2
Description: Table of taxon occurrence data.

File Name: Supplementary Data 3
Description: Clustering vectors from cluster analysis of palaeocoordinate data.

File Name: Supplementary Data 4
Description: Taxon-region presence/absence matrices analysed in this study.

File Name: Supplementary Data 5
Description: Example script for phylogenetic network biogeography analysis.

File Name: Supplementary Data 6
Description: Script for analyses performed within the paper.
